# Dynamic decomposition of spatiotemporal neural signals

**DOI:** 10.1371/journal.pcbi.1005540

**Published:** 2017-05-30

**Authors:** Luca Ambrogioni, Marcel A. J. van Gerven, Eric Maris

**Affiliations:** Radboud University, Donders Institute for Brain, Cognition and Behaviour, Nijmegen, The Netherlands; Duke University, UNITED STATES

## Abstract

Neural signals are characterized by rich temporal and spatiotemporal dynamics that reflect the organization of cortical networks. Theoretical research has shown how neural networks can operate at different dynamic ranges that correspond to specific types of information processing. Here we present a data analysis framework that uses a linearized model of these dynamic states in order to decompose the measured neural signal into a series of components that capture both rhythmic and non-rhythmic neural activity. The method is based on stochastic differential equations and Gaussian process regression. Through computer simulations and analysis of magnetoencephalographic data, we demonstrate the efficacy of the method in identifying meaningful modulations of oscillatory signals corrupted by structured temporal and spatiotemporal noise. These results suggest that the method is particularly suitable for the analysis and interpretation of complex temporal and spatiotemporal neural signals.

## Author summary

In neuroscience, researchers are often interested in the modulations of specific signal components (e.g., oscillations in a particular frequency band), that have to be extracted from a background of both rhythmic and non-rhythmic activity. As the interfering background signals often have higher amplitude than the component of interest, it is crucial to develop methods that are able to perform some sort of signal decomposition. In this paper, we introduce a Bayesian decomposition method that exploits a prior dynamical model of the neural temporal dynamics in order to extract signal components with well-defined dynamic features. The method is based on Gaussian process regression with prior distributions determined by the covariance functions of linear stochastic differential equations. Using simulations and analysis of real MEG data, we show that these informed prior distributions allow for the extraction of interpretable dynamic components and the estimation of relevant signal modulations. We generalize the method to the analysis of spatiotemporal cortical activity and show that the framework is intimately related to well-established source-reconstruction techniques.

This is a *PLOS Computational Biology* methods paper.

## Introduction

Human neocortex has an impressively complex organization. Cortical electrical activity is determined by dynamic properties of neurons that are wired together in large cortical networks. These neuronal networks generate measurable time series with characteristic spatial and temporal structure. In spite of the staggering complexity of cortical networks, electrophysiological measurements can often be properly described in terms of a few relatively simple dynamic components. By dynamic components we mean signals that exhibit characteristic properties such as rhythmicity, time scale and peak frequency. For example, neural oscillations at different frequencies are extremely prominent in electroencephalographic (EEG) and magnetoencephalographic (MEG) measurements and have been related to a wide range of cognitive and behavioral states [1–3]. Neural oscillations have been the subject of theoretical and experimental research as they are seen as a way to connect the dynamic properties of the cortex to human cognition [4–8]. Importantly, an oscillatory process can be described using simple mathematical models in the form of linearized differential equations [9].

In this paper, we introduce a framework to integrate prior knowledge of neural signals (both rhythmic and broadband) into an analysis framework based on Gaussian process (GP) regression [10]. The aim is to decompose the measured time series into a set of dynamic components, each defined by a linear stochastic differential equation (SDE). These SDEs determine a prior probability distribution through their associated GP covariance functions. The covariance function specifies the prior correlation structure of the dynamic components, i.e. the correlations between the components’ activity at different time points. Using this prior, a mathematical model of the signal dynamics is incorporated into a Bayesian data analysis procedure. The resulting decomposition method is able to separate linearly mixed dynamic components from a noise-corrupted measured time series. This is conceptually different from blind decomposition methods such as independent component analysis (ICA) and principal component analysis (PCA) [11, 12] that necessarily rely on the statistical relations between sensors and are not informed by a prior model of the underlying signals. In particular, since each component extracted using the GP-based decomposition is obtained from an explicit model of the underlying process, these components are easily interpretable and can be naturally compared across different participants and experimental conditions.

The GP-based decomposition can be applied to spatiotemporal brain data by imposing a spatial smoothness constraint at the level of the cortical surface. We will show that the resulting spatiotemporal decomposition is related to well-known source reconstruction methods [13–16] and allows to localize the dynamic components across the cortex. The connections between EEG/MEG source reconstruction and GP regression have recently been shown by Solin et al. [17]. Our approach complements and extends their work by introducing an explicit additive model of the underlying neural dynamics.

Through computer simulations and analysis of empirical data, we show that the GP-based decomposition allows to quantify subtle modulations of the dynamic components, such as oscillatory amplitude modulations, and does so more reliably than conventional methods. We also demonstrate that the output of the method is highly interpretable and can be effectively used for uncovering reliable spatiotemporal phenomena in the neural data. Therefore, when applied to the data of a cognitive experiment, this approach may give rise to new insights into how cognitive states arise from neural dynamics.

## Results

In the following, we will show how to construct a probabilistic model of the neural dynamics that captures the main dynamical features of the electrophysiological signals. The temporal dynamics of the neural sources are modeled using linear SDEs, and these in turn determine a series of GP prior distributions. These priors will be used to decompose the signal into several dynamic components with a characteristic temporal correlation structure. Building from the temporal model, we introduce a spatiotemporal decomposition method that can localize the dynamic components on the cortical surface.

### Decomposing a signal using temporal covariance functions

#### Modeling neural activity with stochastic differential equations

**Fig 1:**
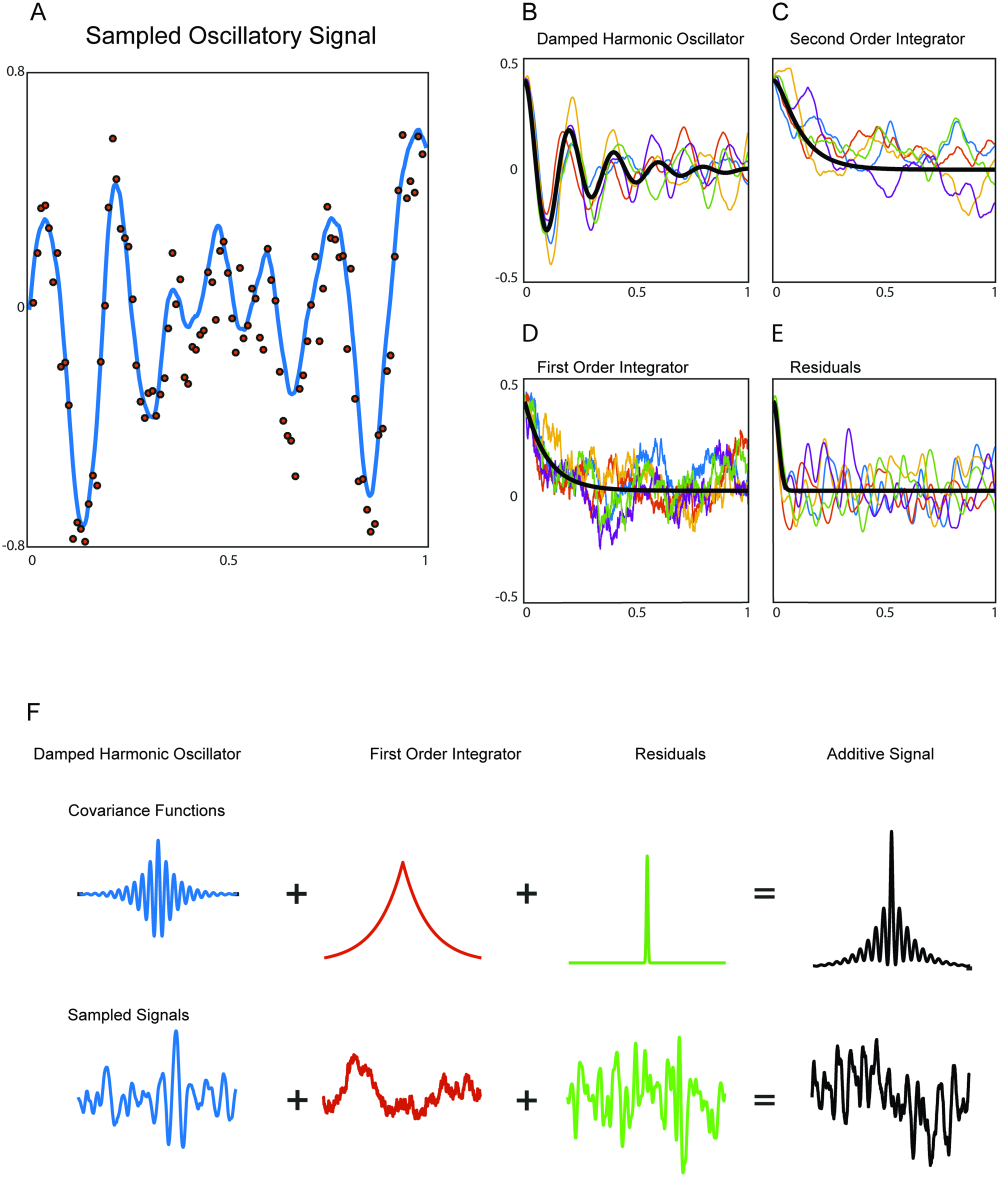
Stochastic processes and covariance functions. A) Example of a continuous-time oscillatory process (blue line) sampled at discrete equally-spaced time points though noise corrupted measurements (red dots). B–E) Samples (colored) and expected values (black) of the stochastic processes. The processes are a damped harmonic oscillator, second order integrator, first order integrator and residuals respectively. The samples start from an excited state and decay back to their respective stationary distribution. F) Illustration of the decomposition of a complex signal’s covariance function into simpler additive components. This corresponds to an additive decomposition of the measured time series. The second order integrator process has been excluded from this panel for visualization purposes.

We consider a single sensor that measures the signal produced by the synchronized subthreshold dynamics of some homogeneous neuronal population. Neural activity is defined for all possible time points but is only observed through discretely-sampled and noise-corrupted measurements *y*_*t*_. We assume the observation noise *ξ*(*t*) to be Gaussian but not necessarily white. In Fig 1A, an example is given of a continuous-time process corrupted by white noise and sampled at regular intervals. Modeling the neural signal as a continuous (rather than a discrete) time series has the advantage of being invariant under changes of sampling frequency and can also accommodate non-equidistant samples.

The prior distribution of the temporal dynamics of the neural activity is specified using linear SDEs. For example, we model the neural oscillatory process *φ*(*t*) using the following equation:

$$\begin{array}{c} \frac{d^{2}}{dt^{2}}\varphi \left(t\right)+b\frac{d}{dt}\varphi \left(t\right)=-\omega_{0}^{2}\varphi \left(t\right)+w\left(t\right)\;. \end{array}$$ (1)

This differential equation describes a damped harmonic oscillator, which responds to input by increasing its oscillatory amplitude. The parameter *b* regulates the exponential decay of these input-driven excitations. The frequency w of these excitations is equal to $\sqrt{\omega_{0}^{2}-\frac{1}{2}b^{2}}$. Clearly, this frequency is only defined for $\omega_{0}^{2}>\frac{1}{2}b^{2}$. For larger values of *b*, the system ceases to exhibit oscillatory responses and is said to be overdamped. These dynamical states are referred to as an oscillator in case $\omega_{0}^{2}>\frac{1}{2}b^{2}$ and an integrator in case $\omega_{0}^{2}<\frac{1}{2}b^{2}$ [18].

We assume the process to be driven by a random input *w*(*t*) (also denoted as *perturbation*). This random function models the combined effect of the synaptic inputs to the neuronal population that generates the signal. Fig 1B shows the expected value (black) and a series of samples (coloured) of the process, starting from an excited state (*φ*(0) = 0.4) and decaying back to its stationary dynamics. Note that the expected value converges to zero whereas the individual samples do not; this is due to the continued effect of the random input. Also, note that the samples gradually become phase inconsistent, with the decay of phase consistency being determined by the damping parameter *b*. Thus, the damping parameter also determines the decay of the temporal correlations.

In general, we model the measured time series as a mixture of four processes, which we will now describe. Of these four, one reflects rhythmic brain activity (i.e., an oscillation), two reflect non-rhythmic brain activity, and one accounts for the residuals:

- *Damped harmonic oscillator.* Oscillations are a feature of many electrophysiological recordings [19, 20], and they are thought to be generated by synchronized oscillatory dynamics of the membrane potentials of large populations of pyramidal neurons [21]. We model the neural oscillatory process as a stochastic damped harmonic oscillator as defined in Eq (1) with damping coefficient $b<\sqrt{2\omega_{0}^{2}}$. This linear differential equation can be obtained by linearizing a model of the neuronal membrane potential that is characterized by sub-threshold oscillations [18].
- *Second order integrator.* We model the smooth non-oscillatory component of the measured time series using an equation of the same form as Eq (1) but in the overdamped state. We will denote this dynamic component as *χ*(*t*). In the overdamped regime, the equation has smooth, non-rhythmic solutions (see Fig 1C). Equations like these emerge by linearizing neuronal models around a non-oscillatory fixed point [18].
- *First order integrator.* Most neurophysiological signals have a significant amount of energy in very low frequencies. We model this part of the signal with a simple first order SDE of which the covariance function decays exponentially. This process captures some of the qualitative features of the measured time series, such as roughness and non-rhythmicity. The model is determined by the following first order SDE:
  $$\begin{array}{c} \frac{d}{dt}\psi \left(t\right)=-c\psi \left(t\right)+w\left(t\right)\;. \end{array}$$ (2)
  The positive number *c* determines the exponential relaxation of the process, i.e. how fast its mean decays to zero after a perturbation. The resulting stochastic process is known as Ornstein-Uhlenbeck process. For a compact neuron this is a good model of the sub-threshold membrane potential under random synaptic inputs [22]. See Fig 1D for some samples of this process.
- *Residuals.* Finally, we account for the residuals *ξ*(*t*) of our model using a process with temporal covariance that decays as $e^{-\frac{t^{2}}{2\delta^{2}}}$, where *δ* is a small time constant. This noise is characterized by short-lived temporal autocorrelations (see Fig 1E). As *δ* tends to zero, the process tends to Gaussian white noise. The temporal covariance of this component was not derived from a stochastic differential equation.

#### From stochastic differential equations to Gaussian processes regression

In our dynamical model, the random input is Gaussian and the dynamics are linear. The linearity implies that the value of the process at any time point is a linear combination of the random input at the past time points. As a consequence, because every linear combination of a set of Gaussian random variables is still Gaussian, the solutions of the SDEs are Gaussian. The Gaussian Process (GP) distribution is the generalization of a multivariate Gaussian for infinitely many degrees of freedom, where the covariance function of the former is analogous to the covariance matrix of the latter. As a zero-mean multivariate Gaussian distribution is fully specified by a covariance matrix, a zero-mean GP *α*(*t*) can be completely determined by its covariance function:

$$\begin{array}{c} k_{\alpha }\left(t,t^{\prime }\right)=\text{cov}\left(\alpha \left(t\right),\alpha \left(t^{\prime }\right)\right) \end{array}$$ (3)

which captures the temporal correlation structure of the stochastic process *α*(*t*). In our case, the covariance function of the dynamical component *φ*(*t*), *χ*(*t*) and *ψ*(*t*) can be obtained analytically from Eqs (1) and (2). This allows to derive a GP distribution for each linear SDE. Moreover, a sum of independent GPs is again a GP, but now with a covariance function that is the sum of the covariance functions of each of its components. This decomposition of the covariance function is exemplified in Fig 1F, which shows the decomposition of the covariance function of a complex signal into several component-specific covariance functions, together with examples of the corresponding dynamic component time series. For visual clarity, the second order integrator component has been omitted from this figure.

With these GPs as prior distributions, we can use Bayes’ theorem for estimating the time course of the dynamic components from the measured time series *y*. In particular, we assume that *y* is generated by the sum of all dynamic components and corrupted by Gaussian noise *ξ*(*t*). The aim is to individually estimate the posterior marginal expectations of *φ*(*t*), *χ*(*t*) and *ψ*(*t*). These marginal expectations are estimates of a dynamic component time course obtained by filtering out from *y* all the contributions of the other components plus the noise.

Since both the prior distributions and the observation model are Gaussian, the posterior distribution is itself Gaussian and its marginal expectations can be computed exactly (see Eq (20) in Materials and methods).

### Spatiotemporal GP-based decomposition

So far, we have shown how SDE modeling of dynamic components can be used for analyzing a neural time series through GP regression. Here, we complement this temporal model by introducing a spatial correlation structure. In this way, we define a full spatiotemporal model. We define the total additive spatiotemporal neural signal as follows:

ρ(x,t)=φ(x,t)+χ(x,t)+ψ(x,t),

where ***x*** denotes a cortical location. Strictly speaking, *ρ*(***x***, *t*) should be a vector field because the neural electrical activity at each cortical point is modeled as an equivalent current dipole. However, for simplicity, we present the methods for the case in which the dipole orientation is fixed and *ρ*(***x***, *t*) can be considered as a scalar field. All formulas for the vector-valued case are given in the supporting information.

#### Modeling spatial correlations

Correlations between different cortical locations can be modeled using a spatial covariance function *s*(***x***, ***x***′). Since the localization of an electric or magnetic source from a sensor array is in general an ill-posed problem, the specification of a prior covariance function is required in order to obtain a unique solution [15]. We do not model the spatial correlation structure directly using spatial SDEs. Instead, we impose a certain degree of spatial smoothness, and this is motivated by the fact that fine details of the neural activity cannot be reliably estimated from the MEG or EEG measurements. This procedure has been shown to reduce the localization error and attenuate some of the typical artifacts of source reconstruction [14, 16].

Modeling the spatial correlations between measurements of neural activity requires a proper definition of distance between cortical locations. The conventional Euclidean distance is likely to be inappropriate because cortical gyri can be nearby according to the Euclidean distance in three-dimensional space, but far apart in terms of the intrinsic cortical geometry that is determined by the synaptic connectivity between grey matter areas. Surface reconstruction algorithms such as Freesurfer [23] allow to map each of the cortical hemispheres onto a sphere in a way that preserves this intrinsic cortical geometry. Building this spherical representation, we can make use of the so-called spherical harmonics. These are basis functions that generalize sines and cosines on the surface of a sphere and are naturally ordered according to their spatial frequency. Using the spherical harmonics we define a spatial covariance function *s*(***x***, ***x***′) between cortical locations, and choose a particular covariance function by discounting high spatial-frequency harmonics. This operation smooths out the fast-varying neural activity and thereby induces spatial correlations. This can be interpreted as a low-pass spatial filter on the cortical surface. The amount of spatial smoothing is regulated by a smoothing parameter *υ* and a regularization parameter λ, where the former controls the prior spatial correlations and the latter the relative contribution of the prior and the observed spatial correlation (see Eqs (23) and (36) in the Materials and methods).

#### Decomposing spatiotemporal signals using separable covariance functions

We combine the spatial and temporal model by making a separability assumption, namely we assume that the covariance between *ρ*(***x***, *t*) and *ρ*(***x***′, *t*′) is given by the product *k*_*ρ*_(*t*, *t*′)*s*(***x***, ***x***′). Using this spatiotemporal GP prior we compute the marginal expectations of the spatiotemporal dynamic components (see Eq (36) in Materials and methods). We refer to this approach as spatiotemporal GP-based decomposition (SGPD).

### Estimating the model parameters

The covariance functions of the dynamic components have parameters that can be directly estimated from the data. Instead of using a full hierarchical model, we estimate the parameters by fitting the total additive covariance function of the model to the empirical auto-covariance matrix of the measured time series using a least-squares approach. This procedure allows to infer the parameters of the prior directly from the data, thereby tuning the dynamical model on the specific features of each participant/experimental condition. Specifically, the parameters of the prior are estimated from the data of all trials, and these parameters in turn determine the GP prior distribution that is used for the analysis of the trial-specific data.

The details of the cost function are described in the Materials and Methods section. Because this optimization problem is not convex, it can have several local minima. For that reason, we used a gradient-free simulated annealing procedure [24] to find a good approximate solution to the global optimization problem.

### Analyzing oscillatory signals using GP-based decomposition: Simulation studies

We conducted three simulation studies to compare the performance of GP-based decomposition with the performance of existing methods. In the first study, we evaluate the ability of the method to recover components from complex spatiotemporal signals. In the second simulation, we evaluate its performance in estimating modulations of oscillatory amplitude. And in the third simulation, we evaluate its performance in localizing the source of an oscillatory amplitude modulation.

#### Recovering components from complex neural signals

Using simulated signals, we evaluated the performance of SGPD in recovering components from mixed oscillatory signals corrupted by temporally and spatially structured noise. We first established the robustness of the GP decomposition method with respect to over-estimation of the number of dynamic components. We did this in a simplified situation where only the temporal dimension is relevant. Next, using spatiotemporal signals, we compared the performance of SGPD with several commonly used decomposition methods. We simulated signals both using a one- and a two-dimensional cortical sheet, with the latter involing non-contiguous spatial profiles. For simplicity, in all these simulations, we assume that the neural activity can be measured directly, thereby obviating the need for a forward model. We will validate GP-based source reconstruction, in which the forward model is an inherent component, in a later paragraph.

In the study evaluating robustness to over-estimation, we simulated signals in the following conditions: (1) two oscillatory processes (5 Hz and 10 Hz) plus an OU process, (2) one oscillatory process (10 Hz) plus an OU process, and (3) a single oscillatory process (10 Hz). In all three conditions, the signals also contained white noise and the total SNR was set equal to 1. In all conditions, we used the temporal GP decomposition with four dynamic components (two damped oscillators, a first order integrator and a residual) to recover the 10 Hz oscillatory process. The performance of the decomposition is extremely similar for all conditions: the median correlations with the ground truth signals were, respectively, 0.947, 0.947 and 0.940 for condition 1,2 and 3. This shows that the method can be very robust to overestimation of the number of dynamic components.

**Fig 2:**
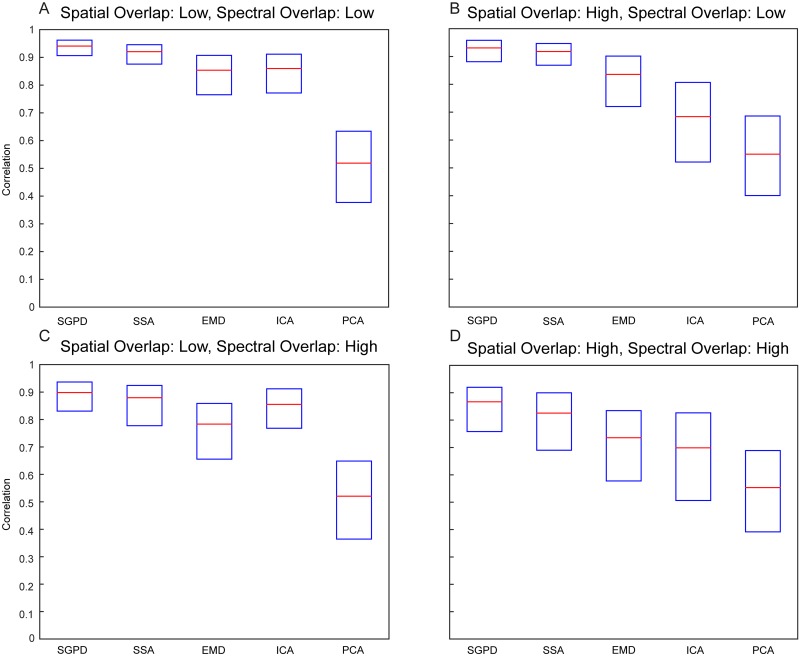
Simulation results on the decomposition of spatiotemporally mixed signals. Performance of SGPD, SSA, EMD, ICA and PCA in recovering an oscillatory signal that was mixed with complex spatiotemporal noise. The performance is quantified as correlation with the ground truth. The red line is the median correlation across trials, the boxes contain correlations between the first and the third quartile. There are four conditions: A) low spectral and low spatial overlap; B) high spectral and low spatial overlap; C) low spectral and high spatial overlap; D) high spectral and high spatial overlap.

In the second simulation, we compared the performance of SGPD with some of the most commonly used signal decomposition techniques: ICA, PCA, empirical mode decomposition (EMD) and singular spectrum analysis (SSA) [11, 25, 26]. All methods were used to extract the time series of a high frequency oscillatory component (10Hz) with a contiguous spatial profile from a mixed spatiotemporal signal. The SGPD was adapted to the one-dimensional case by working with one-dimensional Fourier basis functions (sines and cosines) instead of the two-dimensional spherical harmonics. The oscillatory dynamic component was estimated for all source points, then a single time series was obtained by selecting the source point with the highest variance. EMD and SSA components were extracted separately for each spatial source point and the performance was solely evaluated for the component with highest temporal correlations with the ground truth signal. Performance was quantified as the temporal correlation between the recovered time series and the simulated oscillatory signal (the ground truth). We simulated two oscillatory sources with Gaussian spatial profiles whose centers have coordinates -0.5 mm and 0.5 mm. We manipulated both the spectral and the spatial overlap of the two oscillatory sources. Specifically, the standard deviation of their spatial profiles was either 1 mm (low spatial overlap) or 2 mm (high spatial overlap) and the difference between their peak frequency was either 5 Hz (low spectral overlap) or 2 Hz (high spectral overlap). One of the two oscillatory sources had a peak frequency of 10 Hz, the other source had a lower peak frequency. In all these conditions, the oscillatory sources were surrounded by two noise sources with dynamics governed by an OU process. These noise sources had Gaussian spatial profiles centered at -1.5 mm and 1.5 mm and standard deviation of 1 mm. In total, we generated 600 trials, each with a length of 0.8 s. More details are given in the Methods. Fig 2 shows the correlations between the ground truth signals and the recovered oscillatory components for all listed methods. SGPD performs better than the other methods in all four conditions: high and low spectral overlap combined with high and low spatial overlap. SSA is a good second best, followed by EMD. The performance of ICA depends on the degree of spatial overlap: when the spatial overlap is low, its performance is slightly better than the performance of EMD, but when the spatial overlap is high, its performance is inferior. Finally, in all four conditions, PCA showed the worst performance in recovering the ground truth component.

**Fig 3:**
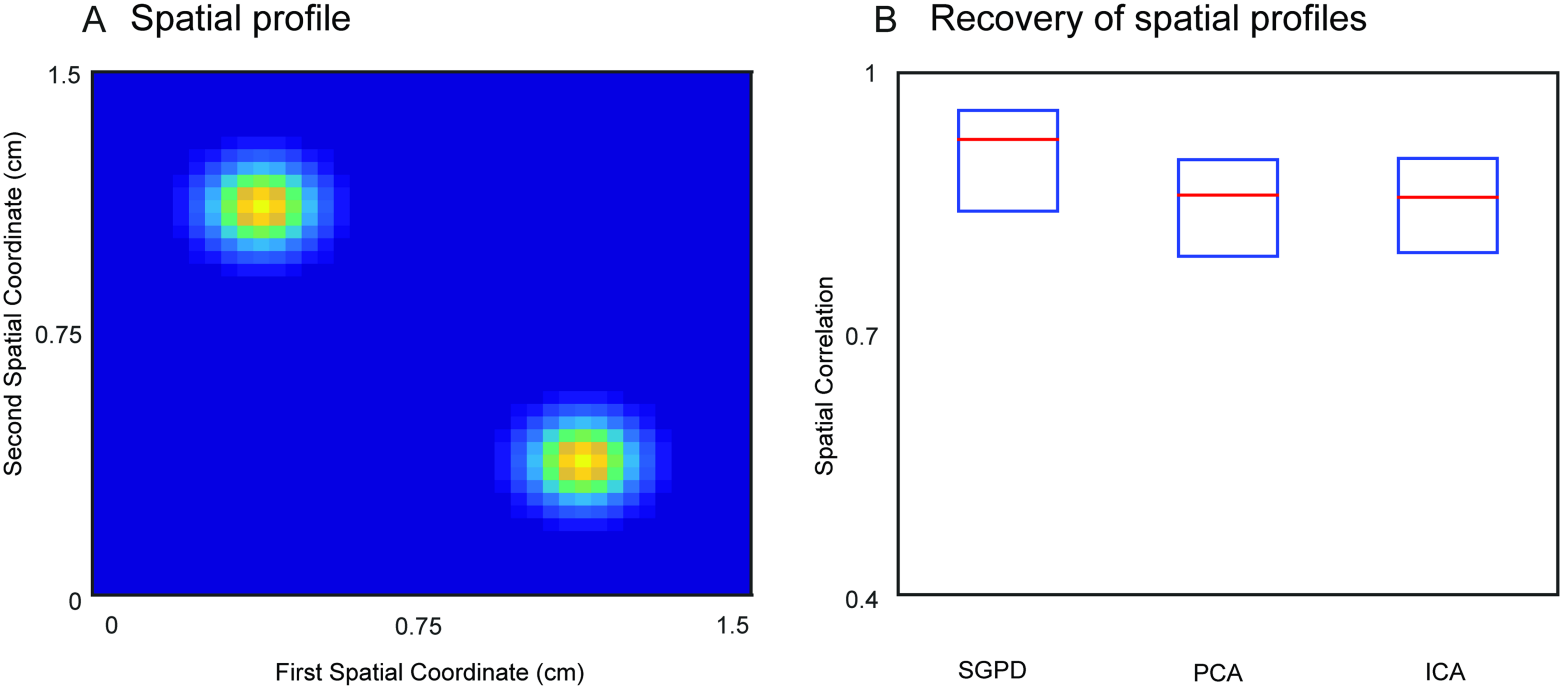
Simulation results on recovery of spatial profiles of non-contiguous dynamic components. Performance of SGPD, ICA and PCA in recovering an oscillatory signal with a non-contiguous spatial profile. A) Ground truth spatial profile of the dynamic component. B) Performance of SGPD, PCA and PCA in recovering the spatial profile of a dynamic component with bi-modal spatial profile on a two-dimensional cortical sheet. The performance is quantified as spatial correlation with the ground truth.

Our third simulation, is motivated by the fact that cortical spatiotemporal components can have a non-contiguous spatial profile. One of the best known examples is the default mode network, a cortical network that spans several brain areas [27]. In our simulation, we investigate the capacity of SGPD to recover the spatial profile of a simulated non-contiguous dynamic component. Since component analysis methods such as ICA and PCA are by far the most widely used for recovering the spatial profile of functional networks, we limited our comparison to those two methods. The source model is a two-dimensional flat cortical sheet. For each simulated trial, we generated an oscillatory source (peak frequency: 10 Hz) with a spatial profile that was the sum of two bivariate isotropic Gaussian functions with centers at coordinate pairs (1, 1) mm and (−1, −1) mm and with standard deviation equal to 0.2 mm. This spatial profile is shown in Fig 3A. These two peaks of activity are practically non-contiguous, being separated by five standard deviations. The oscillatory signal was contaminated by an OU process with a spatial profile that is also the sum of two bivariate isotropic Gaussian functions with centers at coordinate paris (1, −1) mm and (−1, 1) and with standard deviation equal to 0.8 mm. Finally, the data was corrupted with spatiotemporal white noise. Except for the presence of a single oscillatory source, the details of all the signals are identical to the previous simulation. We evaluated the performance of SGPD in recovering the spatial pattern of the oscillatory source. As a comparison, the figure also reports the spatial correlations of the estimates obtained using PCA and temporal ICA. In this latter case, we applied the ICA algorithm on the temporally-concatenated data matrix. From every resulting decomposition, we selected the component with the highest temporal correlation with the ground truth oscillation. Next, for both ICA and PCA, we obtained trial-by-trial estimates of the spatial maps by least squares fitting of the selected component’s time course on the trial specific data. The SGPD had a median correlation of 0.9 while PCA and ICA had a lower median correlation of 0.77 and 0.78 respectively. Fig 3B shows the spatial correlations between the ground truth spatial profile and the estimate obtained using SGPD, PCA and ICA.

#### Estimating modulations in oscillatory amplitude

Modulations in oscillatory amplitude have been related to many cognitive processes. For example, in tasks that require attentional orienting to some part of the visual field, alpha oscillations are suppressed over the corresponding brain regions [28, 29]. Because the spectral content of electrophysiological measurements is almost always broadband, when there is an interest in oscillations, it makes sense to isolate these oscillations from the other components of the measured time series. The resulting procedure involves a separation of the oscillatory components of interest from the interfering non-rhythmic components. In the GP-based decomposition framework, this separation can be achieved by modeling both the oscillatory component *φ*(*t*) and the interfering processes. We use the symbol ***m***_*φ*|*y*_ for the marginal expectation of the process *φ*(*t*) at the sample points. The average amplitude can be obtained from ***m***_*φ*|*y*_ by calculating its root mean square deviation:

$$A=\sqrt{\frac{1}{N}\sum_{j}{\left({\left[m_{\varphi |y}\right]}_{j}-\bar{m}\right)}^{2}}$$ (4)

with $\bar{m}=\frac{1}{N}\Sigma_{i}{\left[m_{\varphi |y}\right]}_{i}$.

Here, we compared the sensitivity of the GP method with DPSS multitaper spectral estimation [30], a widely used non-parametric technique. In the simulation study, the methods had to estimate a simulated experimental modulation of the amplitude of a 10 Hz oscillatory process. For each of two conditions, we generated oscillatory time series from a non-Gaussian oscillatory process. The oscillatory time series was then corrupted by Ornstein–Uhlenbeck (OU) noise and white noise. The simulation design involved 16 levels that covered an amplitude modulation range from 15% to 60% in equidistant steps. For each level, per experimental condition, we generated 150,000 trials of 2 s. The effect size was defined as follows:

$$\begin{array}{c} f=\frac{\left\langle A_{1}\right\rangle -\left\langle A_{2}\right\rangle }{\text{var}(A)}\;, \end{array}$$ (5)

where 〈*A*_*j*_〉 is the mean oscillatory amplitude in the j-th experimental condition and var (*A*) is its variance. Mean and variance were calculated over the trials.

**Fig 4:**
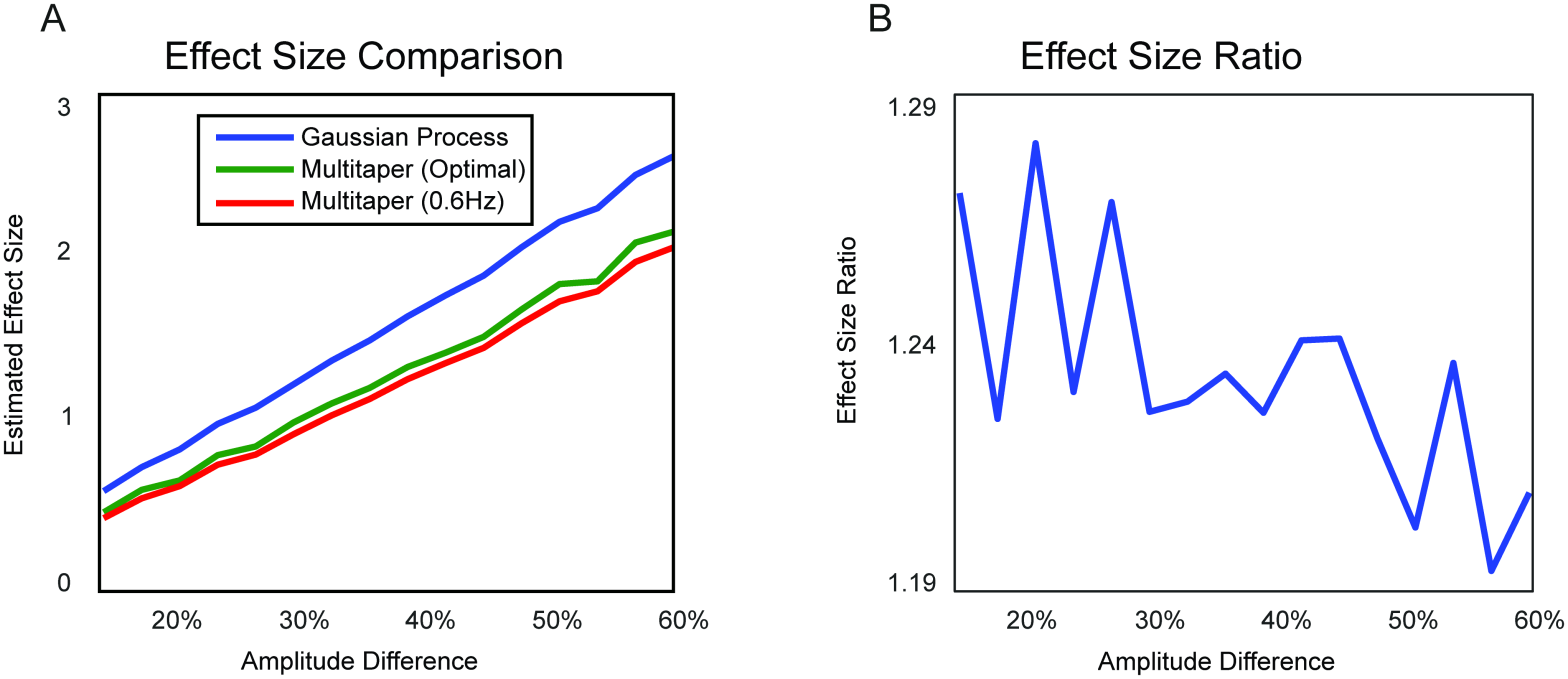
Simulation results on the estimation of modulations in oscillatory amplitude. A) Effect size of temporal GP and DPSS multitaper spectral analysis as function of mean percentage amplitude difference between simulated conditions. The parameters of the temporal GP-based decomposition (blue line) were estimated from the raw simulated time series. The spectral smoothing of the multitaper method (green line) was chosen for each to maximize the effect size. The red line is the effect size for a multitaper method with constant spectral smoothing of 0.6 Hz. B) Effect size ratio between temporal GP and (optimized) multitaper method as function of the mean amplitude difference between conditions.

The GP method does not have free parameters, since the parameters of the covariance functions are estimated from the data. In contrast, the spectral smoothing of a multitaper analysis is determined by the number of tapers, which is a parameter that can be chosen freely. We selected the number of tapers that maximizes the effect size in order not to bias the evaluation in favor of the GP method. In addition, we reported the effect sizes for the multitaper analysis with a fixed smoothing of 0.6 Hz. Fig 4A shows the effect sizes for the GP and the multitaper method as a function of the true between-condition amplitude difference. The Gaussian process consistently outperforms the non-parametric method. Fig 4B shows the ratio between the GP and the optimal multitaper effect size as a function of the true amplitude difference. Here, we can see that the superior performance is more pronounced when the amplitude difference is smaller, corresponding to a situation with a lower signal-to-noise ratio.

#### Localizing the source of an oscillatory amplitude modulation

We now investigate how SGPD compares to a state-of-the-art existing methods with respect to the spatial localization of an oscillatory amplitude modulation in the presence of noise sources with both spatial and temporal structure. We compare our method to the Harmony source reconstruction technique [16], which has been shown to outperform several commonly used linear source reconstruction methods. For this, we set up a simulation study in which the performance was evaluated by the extent to which a spatially focal amplitude modulation could be detected.

**Fig 5:**
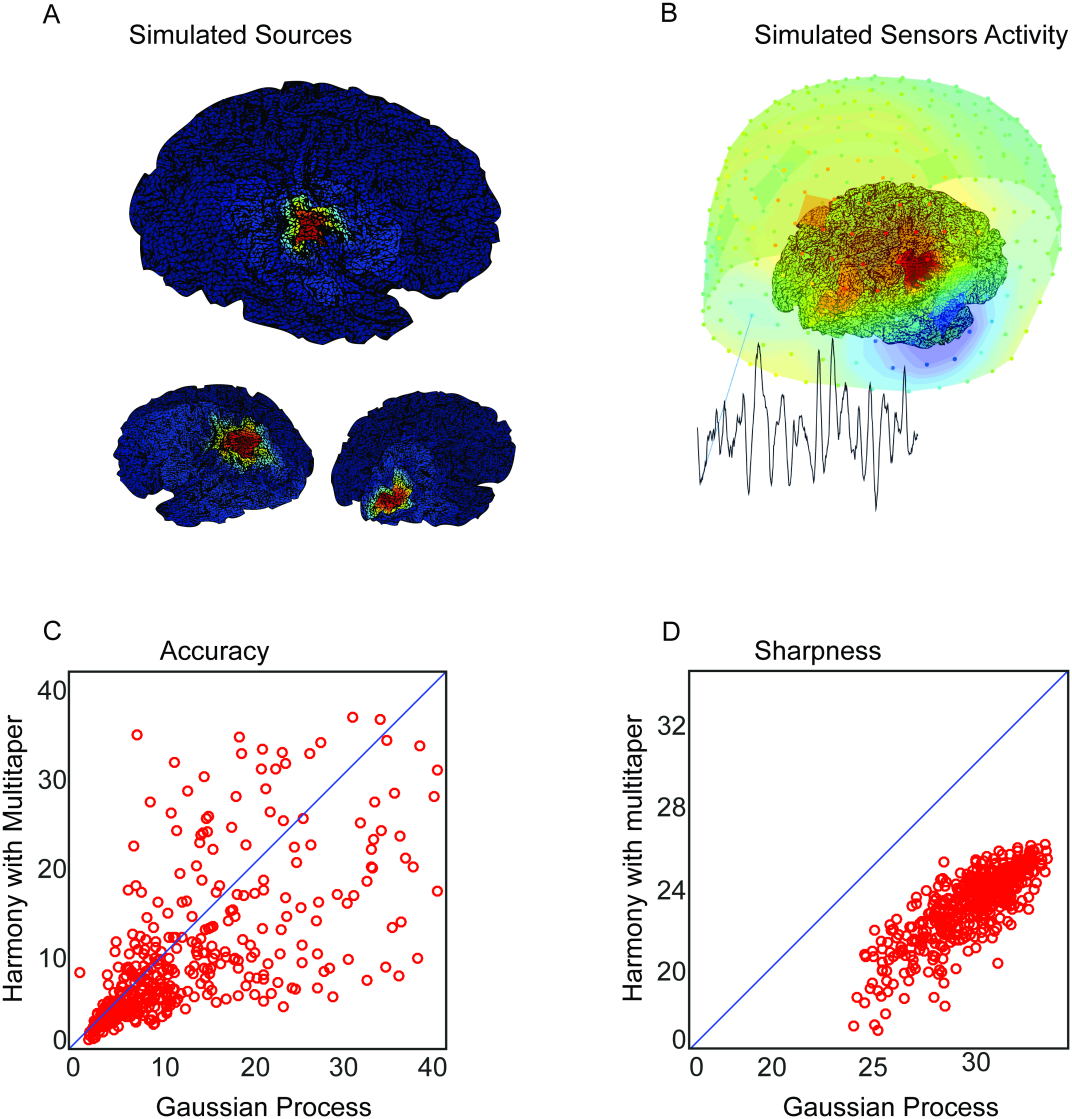
Simulation results on localizing the source of an oscillatory amplitude modulation. A) Spatial maps of the simulated brain sources. The left map shows the spatial extent of the amplitude-modulated source while the two right maps show the interfering sources. The dipole orientation was set to be orthogonal to the mesh surface. B) Visualization of sensor activity as a mixing of the three sources. The dots represent MEG sensors. The color of the dots show the sign (red for positive and blue for negative) together with the magnitudes. The time series was taken from an occipital sensor. C) Scatter plot of the accuracy of SGPD and Harmony. The index was computed by dividing the total reconstructed effect within the amplitude-modulated cortical patch by the sum of total effects in the non-modulated patches. D) Scatter plot of the sharpness of SGPD and Harmony. The sharpness index was obtained by dividing the total reconstructed effect within the amplitude-modulated cortical patch by the total effect elsewhere. For the purpose of visualization, in both scatterplots, we excluded some outliers (> 5 × median). These outliers arise when the denominator of one of the indices becomes too small. The outliers have been removed from the figure but they were involved in the calculation of the medians for the two methods.

We modeled the brain activity as generated by three cortical patches, each with a constant spatial profile and a time course generated in the same way as in the single sensor simulation. The patches had a radius of approximately one centimeter and were localized in the right temporal, right occipital, and left parietal cortex (Fig 5A). All three patches exhibited oscillatory activity, but the one in the right temporal lobe had an amplitude that was modulated by the simulated conditions. The source activity was projected to the sensors by a forward model that was obtained using a realistic head model [31]. The sensor level activity for the first trial is shown in Fig 5B. The regularization parameter l of both Harmony and SGPD were identified using leave-one-out cross validation [32], while the smoothing parameter *υ* was set by hand and had the same value of 3 in both models. The spectral smoothing of the DPSS multitaper spectral estimation was set to 0.6 Hz. The value was chosen because, on average, this gave the highest effect size of the amplitude modulation.

We assessed the quality of the reconstructed effects using two indices, one for accuracy and one for sharpness. The accuracy index is obtained by dividing the estimated effect in the center of the amplitude-modulated patch (more specifically, the sum over the points in a sphere with 1 cm radius) by the maximum of the estimated effects in the centers of the other two patches (again, by summing over the points in a sphere). The accuracy index will be high if it localizes the effect in the right patch but not in the interfering ones. The sharpness index evaluates how much the effect maps are focused around the center of the effect. It is computed by dividing the summed estimated effect in the center of the amplitude-modulated patch by the summed estimated effect outside that region. Fig 5C & 5D show the results of the simulation. Each disc in the scatter plot represents the outcome of SGPD and Harmony for a single simulation. The median accuracy and sharpness were respectively 33% and 28% higher for SGPD as compared to the Harmony approach.

### Gaussian process analysis of example MEG data

**Fig 6:**
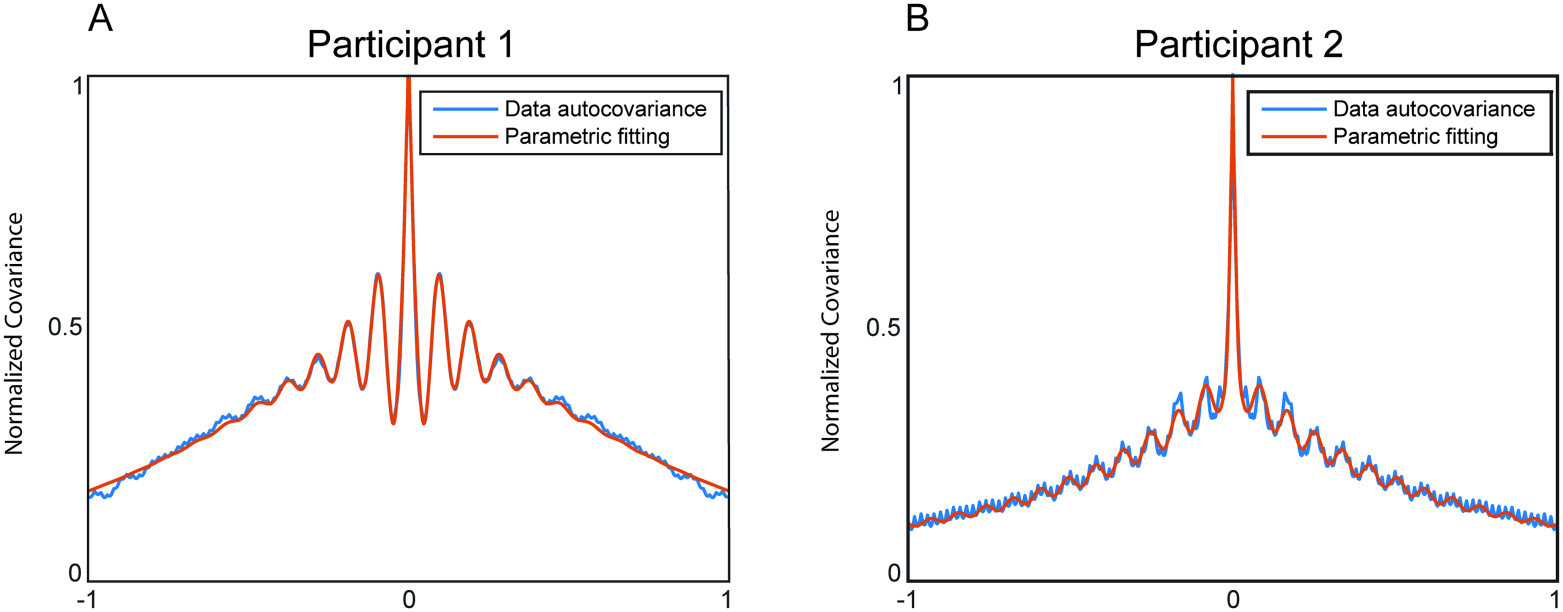
Estimation of the model covariance functions. Parametric fit of the MEG auto-covariance functions of Participant 1 and Participant 2. The red lines refer to the estimated parametric model and the blue lines reflect the empirical auto-covariance of the measured time series. A single auto-covariance was obtained from the multi-sensor data by performing a principal component analysis and averaging the empirical auto-covariance of the first 50 components, weighted by their variance. The parameters of the model were estimated using a least-squares simulated annealing optimization method. The graphs have been scaled between 0 and 1 by dividing them by the maximum of the individual empirical auto-covariance.

We tested the temporal GP-based decomposition on an example MEG dataset that was collected from 14 participants that performed a somatosensory attention experiment [33]. We will use this dataset for different purposes, and start by using it for evaluating the performance of our parameter estimation algorithm. Fig 6 shows the empirical auto-covariance functions and the least squares fit for two participants. To make them comparable, we normalized these auto-covariance functions by dividing them by their variance. The fitted auto-covariance functions capture most features of the observed auto-covariance functions. The comparison shows some individual differences: First, Participant 1 has a higher amplitude alpha signal relative to the other dynamic components, but the correlation peaks are separated only by about three cycles. Second, the auto-covariance of Participant 2 is dominated more by a signal component with a high temporal correlation for nearby points, and the rhythmic alpha component decays much more slowly. The latter is a signature of a longer phase preservation.

We quantified the goodness-of-fit as the normalized total absolute deviation from the model:

$$\begin{array}{c} g=\frac{\sum_{i,j}\left|c_{ij}-k\left(t_{i},t_{j}\right)\right|}{\sum_{i,j}\left|c_{ij}\right|}\;, \end{array}$$ (6)

where *c*_*ij*_ is the empirical auto-covariance between $y_{t_{i}}$ and $y_{t_{j}}$, and *k*(*t*_*i*_, *t*_*j*_) is the auto-covariance predicted by our dynamical model. We evaluated the goodness-of-fit by computing this deviation measure for each participant. The median goodness-of-fit was 0.06, meaning that the median deviation from the empirical auto-covariance was only 6% of the sum of its absolute values. The goodness-of-fit for the two example participants one and two in Fig 6 are 0.04 and 0.02, respectively.

**Fig 7:**
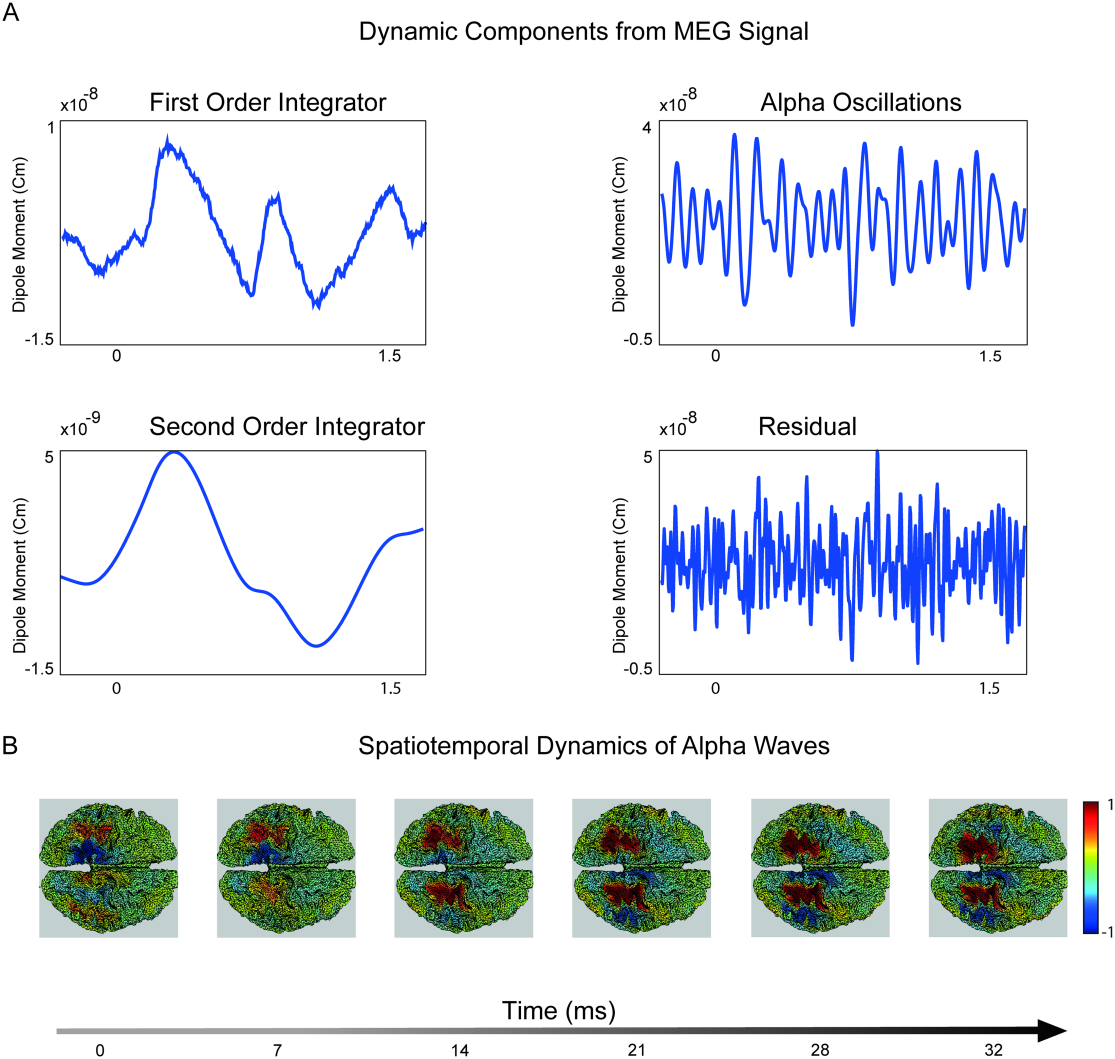
Estimated dynamic components. Reconstructed source-level neural activity of Participant 1. A) Reconstructed time series of the four dynamic processes localized in a right parietal cortical vertex. B) Reconstructed spatiotemporal dynamics of alpha oscillations along the *x* axis. This choice of axis is arbitrary and has been chosen solely for visualization purposes. The source-reconstructed activity has been normalized by dividing it by the maximum of the absolute of the spatiotemporal signal.

Next, we inspect the reconstructed spatiotemporal dynamic components obtained from the resting state MEG signal of Participant 1 (with auto-covariance as shown in Fig 6A), as obtained by SGPD. Fig 7A shows an example of time courses of the dynamic components for an arbitrarily chosen cortical vertex situated in the right parietal cortex. The first order integrator time series (upper-left panel) tends to be slow-varying but also exhibits some fast transitions. The second order integrator (lower-left panel) is equally slow but smoother. In this participant, the alpha oscillations, as captured by the damped harmonic oscillator, are quite irregular (upper-right panel), and this is in agreement with its covariance function (see Fig 6A). Finally, the residuals (lower-right panel) are very irregular, as is expected from the signal’s short-lived temporal correlations. Fig 7B shows an example of the spatiotemporal evolution of alpha oscillations for a period of 32 milliseconds in a resting-state MEG signal. For the purpose of visualization, we only show the value of the dipole along an arbitrary axis. The pattern in the left hemisphere has a wavefront that propagates through the parietal cortex. Conversely, the alpha signal in the right hemisphere is more stationary.

### Attention-induced spatiotemporal dynamics of oscillatory amplitude

Next, we applied the SGPD source reconstruction method to the example MEG data that were collected in a cued tactile detection experiment. Identifying the neurophysiological mechanisms underlying attentional orienting is an active area of investigation in cognitive neuroscience [8, 28, 33, 34]. Such mechanisms could involve neural activity of which the spatial distribution varies over time (i.e., neural activity with dynamic spatial patterns), and GP source reconstruction turns out to be highly suited for identifying such activity, as we will demonstrate now.

In the cued tactile detection experiment an auditory stimulus (high or low pitch pure tone) cued the location (left or right hand) of a near-threshold tactile stimulus in one-third of the trials. This cue was presented 1.5 s before the target. The remaining two-thirds of the trials were uncued. In the following, we compare the pre-target interval between the cued and the uncued conditions in terms of how the alpha amplitude modulation develops over time. In the analysis, we made use of the fact that the experiment involved two recording sessions, separated by a break. We explored the data of the first session in search for some pattern, and then used the data of the second session to statistically test for the presence of this pattern. Thus, the spatiotemporal details of the null hypothesis of this statistical test were determined by the data of the first session, and we used the data of the second session to test it.

**Fig 8:**
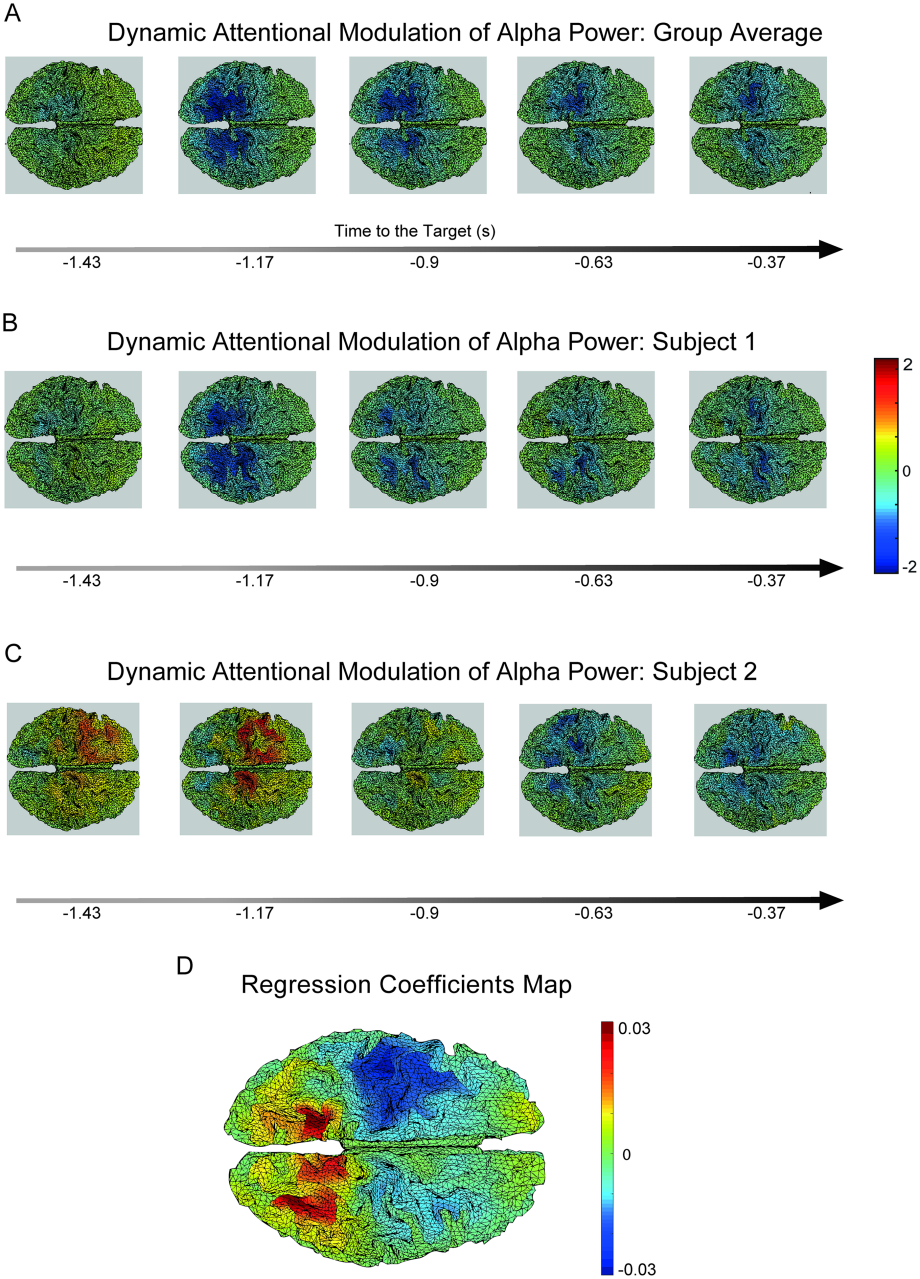
Caudal-to-rostral progression of alpha amplitude attentional modulation. A) Group average of alpha amplitude attentional modulation as function of time. B,C) Alpha amplitude attentional modulation for participants 1 and 2, respectively. D) Spatial map obtained by computing the slope of the average alpha difference between cued and non-cued conditions as a function of time for each cortical vertex.

Fig 8A shows the group-averaged alpha amplitude modulation as a function of time. An amplitude suppression for the cued relative to the uncued condition originates bilaterally in the parietal cortex and gradually progresses caudal to rostral until it reaches the sensorimotor cortices. The time axes are expressed in terms of the distance to the target. Similar patterns can be seen in individual participants (see Fig 8B & 8C for representative participants 1 and 2). Participant 1 has a suppressive profile that is almost indistinguishable from the group average. On the other hand, participant 2 shows an early enhancement of sensorimotor alpha power accompanied by a parietal suppression, and the latter then propagates forward until it reaches the sensorimotor areas. Thus, in the grand average and in most of the participants, there is a clear caudal-to-rostral progression in the attention-induced alpha amplitude suppression. We characterized this progression by constructing cortical maps of the linear dependence (slope) between latency and amplitude modulation. The group average of the slope maps for the first session is shown in Fig 8D. This figure shows that the posterior part of the brain has positive slopes, reflecting the fact that the effect tended to become less negative over time. Conversely, the sensorimotor regions have positive slopes, reflecting the fact that the effect tended to become more negative over time.

To evaluate the reliability of this pattern, we build on the reasoning that, if this pattern in the slope map is due to chance, then it must be uncorrelated with the slope map for the second session. To evaluate this, for every participant, we calculated the dot product between the normalized slope maps for the two sessions and tested whether the average dot product was different from zero.

The one-sample t-test showed that the effect was significantly different from zero (*p* < 0.05), supporting the claim that the caudal-to-rostral progression in the attention-induced alpha amplitude suppression is genuine. Thus, we have shown that, during the attentional preparation following the cue, the alpha modulation progresses from the parietal to the sensorimotor cortex.

## Discussion

In this paper, we introduced a new signal decomposition technique that incorporates explicit dynamical models of neural activity. We showed how dynamical models can be constructed and integrated into a Bayesian statistical analysis framework based on GP regression. The resulting statistical model can be used for decomposing the measured time series into a set of temporal or spatiotemporal dynamic components that reflect different aspects of the neural signal. A spatiotemporal version of the decomposition method was obtained by decomposing the spatial configuration of cortical neural signals in spherical harmonics.

We validated our method using simulations and real MEG data. We performed three simulation studies in which we demonstrated the superior performance of our method for three different applications: the recovery of a component from a complex neural signal, the estimation of a modulation in oscillatory amplitude, and the localization of the source of an oscillatory amplitude modulation. For the first application, we showed that, under a broad range of conditions, our method provided a more accurate recovery than four popular signal decomposition methods: ICA, PCA, EMD and SSA. For the second application, we demonstrated that our method can be used for quantifying changes in oscillatory amplitude between experimental conditions. We showed that, when the oscillatory signal is corrupted by temporally structured noise, our method improves on multitaper non-parametric spectral estimation. Finally, for the third application, we demonstrated that SGPD leads to sharp and precise localization of dynamic components on the cortical surface. In particular, we showed that, in the presence of spatially and temporally structured noise, SGDP localizes amplitude modulations more accurately than Harmony, a related method that does not make use of the temporal decomposition of the signal of interest [16]. Lastly, using the spatiotemporal decomposition on real MEG data from a somatosensory detection task, we demonstrated its usefulness by identifying an intriguing anterior-to-posterior propagation in the attention-induced suppression of oscillatory alpha power.

### Generality, limitations, and robustness

Although we used a specific set of SDEs, the method is fully general in that it can be applied to any linearized model of neuronal activity. Therefore, it establishes a valuable connection between data analysis and theoretical modeling of neural phenomena. For example, neural masses models and neural field equations (see, e.g. [35]) can be linearized around their fixed points and the resulting SDEs form the basis for a GP analysis that extracts the theoretically defined components. Furthermore, the GP-based decomposition could be used as an analytically solvable starting point for the statistical analysis of non-linear and non-Gaussian phenomena through methods such as perturbative expansion, where the initial linear Gaussian model is corrected by non-linear terms that come from the Taylor expansion of the non-linear couplings between the neural activity at different spatiotemporal points [36].

The method’s limitations pertain to the model’s prior assumptions. Our prior model is based on linear stochastic differential equations that cannot account for the complex non-linear effects that are found in both experimental [37, 38] and modeling work [39–41]. In addition, our prior model assumes a homegeneous spatial correlation structure that solely depends on the distance between cortical locations. Clearly, this correlation struction does not account for the rich connectivity structure of the brain [42–45]. Nevertheless, the method has some robustness against the violations of the underlying assumptions. This robustness follows from the fact that the model specifies the prior distribution but does not constrain the marginal expectations to have a specific parametric form. The temporal prior affects the estimation of a dynamic component to a degree that depends on the ratio between its variance and the cumulative variance of all other components. Specifically, the smaller the prior variance of a component relative to the combined variance of all the others, the more the pattern in the prior covariance matrix will affect the posterior. Since we estimate all these prior variances directly from the measured time series, our method is able to reconstruct complex non-linear effects in components that have a relatively high SNR while it tends to “linearize” components with low SNR. As a consequence, the more pronounced the non-linear effects in the observed signal, the more these will be reflected in the posterior, gradually dominating the linear structure imposed by the prior. Importantly, because our temporal prior is based on a larger data set, it will be adequate, on average, over all epochs while still allowing strong components in individual epochs to dominate the results.

The situation is similar but not identical for our spatial prior. Contrary to our temporal prior, this spatial prior is not derived from an empirically fitted dynamical model but on the basis of our prior belief that source configurations with high spatial frequencies are unlikely to be reliably estimated from MEG measurements. Since the problem of reconstructing source activity from MEG measurements is generally ill-posed, the choice of the spatial prior will bias the inference even for very high SNR. Nevertheless, it has been shown that the discounting of high spatial frequencies leads to reduced localization error and more interpretable results [16].

### Connections with other methods

The ideas behind the GP-based decomposition derive from a series of recent developments in machine learning, connecting GP regression to stochastic dynamics [46, 47]. The approach is closely connected with many methods in several areas of statistical data-analysis such as signal decomposition, blind source separation, spectral analysis and source reconstruction. We will now review some of these links, focusing on methods that are commonly used in neuroscience.

#### Signal decomposition

In the neuroscience literature, the most widely used signal decomposition techniques are a class of methods known as *blind source separation*. The two best known members of this class are PCA and ICA, which have been extended in several different ways [11, 12, 48–52]. ICA and PCA rely on the statistical properties of multi-sensor data (maximum variance for PCA and statistical independence for ICA) and produce components whose associated signals are linear combinations of the sensor-level signals. The main difference between PCA and ICA is that the former only relies on second order statistics (correlations between sensors) while the latter exploits higher order statistics such as skewness and kurtosis.

Whereas GP-based decomposition depends on a specific model of the neural signal, neither PCA nor ICA makes use of prior knowledge of the component-level signals. Furthermore, both PCA and ICA require matrix-valued time series data, whereas GP-based decomposition can be applied to a single univariate time series. It is important to note that GP-based decomposition is not a tool for separating statistically independent or uncorrelated components. Instead, its goal is to decompose the measured signal into several processes characterized by different autocorrelation structures. Hence, the method does not discriminate between two independent processes generated by two sources with the same dynamics, such as a frontal and an occipital alpha oscillation. Therefore, the GP-based decomposition is complementary to blind source separation. In fact, the latter can be used to extract interesting temporal and spatiotemporal patterns from the dynamic components obtained from GP-based decomposition.

There also exists a class of signal separation methods that *can* be applied to a single univariate time series. Two members of this class are EMD [25] and SSA [26]. The aim of EMD is to identify meaningful components from a mixed signal without resorting to formal assumptions about these components or an explicit set of basis functions [25]. The components extracted using EMD are referred to as *intrinsic mode functions* (IMF). The absence of formal assumptions does not imply that the method is not biased towards a particular type of component. In fact, by identifying local peaks and troughs, the EMD algorithm effectively searches for oscillatory components. SSA is based on the singular value decomposition (SVD) of the so called trajectory matrix, whose columns are time-lagged copies of the original time-series [26]. The singular vectors of the trajectory matrix are often denoted as *empirical orthogonal functions* and can be seen as a set of temporal signal components. The components extracted using SSA solely depend on the temporal autocorrelation of the time series. Therefore, SSA only uses second order statistics. The main difference between GP-based decomposition and the blind decomposition methods SSA and EMD is that the former makes explicit formal assumptions about the temporal structure in the components in its prior distribution. Specifically, the GP-based decomposition assumes a prior model that is linear and stationary while both EMD and SSA do not assume neither the stationary nor the linearity of the process that generated the components. Although GP-decomposition requires an initial modeling effort, which is not required by SSA and EMD, this effort pays off in the simpler interpretation and comparability of the resulting dynamic components. In fact, the components obtained through SSA and EMD are not labeled and their number is not constant. Consequently, components obtained from different trials/participants are not directly comparable.

So far, we have only discussed alternative decomposition methods that exploit either the temporal or the spatial features of the data. Truly spatiotemporal signal components can be obtained with a straightforward multivariate generalization of SSA, often denoted as multivariate SSA (M-SSA) [53]. M-SSA components can be obtained as eigenvectors of the lagged cross-covariance matrix. Since this eigendecomposition scales quadratically with the product between maximal time lag and number of spatial points, it is difficult to apply this method to large spatiotemporal arrays. Another widely used and intrinsically spatiotemporal decomposition approach is dynamic mode decomposition (DMD) [54]. This method assumes that the spatial pattern at each time point is a linear combination of the spatial pattern at the previous time point. The method involves a state transition matrix, which is usually assumed to be constant during the analyzed epoch. Signal components, also called *dynamical modes*, are obtained by eigendecomposition of the state transition matrix. Similar to EMD and (M-)SSA, DMD does not uses a separate dynamical model of each component and, consequently, the resulting dynamical modes do not have natural labels.

The characteristics of the different signal decomposition methods are summarized in Table 1.

**Table 1:** Comparison of signal decomposition methods.

	GP	PCA	ICA	EMD	(M-)SSA	DMD
Produces labeled components	yes	no	no	no	no	no
Can work on single sensor data	yes	no	no	yes	yes	yes
Combines multiple sensors	yes	yes	yes	no	yes	yes
Combines multiple time points	yes	no	no	yes	yes	yes
Assumes stationarity and linearity of the time series	yes	no	no	no	no	yes
Only uses second order statistics	yes	yes	no	no	yes	yes

From an applications point of view, the most relevant aspect of a signal decomposition method is its recovery of a signal component that is mixed with other components in the observed signal. In simulations that involved signal components with different temporal autocorrelation structures, we showed that that the goodness-of-recovery of our GP-based decomposition was superior to PCA, ICA, EMD and SSA. Importantly, this superiority was maintained across different levels of spatial and spectral overlap between signal and noise.

#### Spectral analysis

In the Results, we have demonstrated that the GP-based decomposition can be profitably used to estimate amplitude modulations in an oscillatory signal, which is an important application of spectral analysis. There are two classes of spectral analysis methods: parametric and non-parametric [30]. Non-parametric methods mostly rely on the discrete Fourier transform applied to a tapered signal, as for example in DPSS multitaper spectral estimation [30, 55]. These methods are non-parametric because they do not explicitly model the process that generates the signal.

Parametric methods *do* depend on an explicit model, and typically this is an autoregressive (AR) model [56, 57]. AR models are closely related to GPs as they are typically formulated as discrete-time Gaussian processes driven by stochastic *difference* equations. In this sense, the GP prior distributions are continuous-time versions of an AR process. However, the usual AR approach to spectral estimation differs from our approach. AR models are usually parametrized in terms of a series of model coefficients that determine the statistical dependencies between the present value of a signal and its past values. These model coefficients are related to the inverse of the impulse response function in our approach (see Materials and methods for a description of the impulse response function). Spectral analysis based on AR models has the disadvantage that one has to estimate a very large number of parameters. This flexibility in the analytic form of the AR model is required as the spectrum is obtained from the model coefficients. Compared to AR modeling, the GP-based decomposition model is much more constrained by the underlying theory, having an explicit additive structure with few parameters for each dynamic component. Specifically, the additive structure allows to isolate the spectrum of a component of the signal from other nuisance components (e.g. the spectrum of an oscillation from the background pink noise). The rigidity of the model is compensated by the fact that the oscillatory amplitude is not obtained from the fitted model covariance function. Instead, it is computed from the marginal expectation of the oscillatory component, which is obtained by applying Bayes’ rule.

In our simulation study, we compared the temporal GP-based decomposition to DPSS multitaper spectral estimation. We showed that, when the oscillatory signal of interest is corrupted by temporally structured noise, our method gives better quantification of changes in oscillatory amplitude. The superiority of our method is probably explained by its ability to learn the features of the signal and the noise from the data.

#### Source reconstruction

A general framework for GP source analysis has recently been introduced [17]. In this work, the authors show that several well-known source reconstruction methods are special cases of GP regression with appropriate covariance functions. In particular, the spatial filter of techniques such as minimum norm estimation [13] and exact Loreta [14] are obtained as a discretization of a spatial GP analysis with an appropriate spatial covariance function. The authors also introduced a general framework for GP spatiotemporal analysis using separable covariance functions designed to localize averaged neural activity (e.g. evoked fields). This GP spatiotemporal source reconstruction is formally similar to several other spatiotemporal source reconstruction methods [58–61].

Our approach improves on this work by introducing informed temporal covariance functions that explicitly model the temporal dynamics of the ongoing neural signal. The additive structure of the temporal covariance function allows to individually source localize signal components with different dynamic properties. The spatial configuration of these components are analyzed in the spherical harmonics domain, as this greatly reduces the dimensionality of the source space. As shown in the Materials and Methods section, the resulting spatial filter is closely related to the Harmony source reconstruction method [16]. However, despite this similarity in spatial filters, our simulation study shows that SGPD leads to more accurate source reconstructions than spatial localization alone.

### Benefits of GP-based decomposition for cognitive neuroscience

In our simulation studies, we demonstrated the superior performance of GP-based decomposition for three different applications: the recovery of a component from a complex neural signal, the estimation of a modulation in oscillatory amplitude, and the localization of the source of an oscillatory amplitude modulation. In addition, this method is also particularly suited for data-driven exploration of complex spatiotemporal data as it decomposes the signal into a series of more interpretable dynamic components. As a demostration of this, we used the SGPD to investigate the modulation of alpha oscillations associated with attentional preparation to a tactile stimulus. Several previous works demonstrated that alpha amplitude is reduced prior to a predicted stimulus [28, 33, 34]. These amplitude modulations have been associated to modality specific preparatory regulations of the sensory cortices [7, 34, 62–64]. While the attentional role of alpha oscillations in the primary sensory cortices is well established, it is still unclear how this generalizes to supramodal areas. Although the parietal cortex is known to play a role in the top-down control of attention [65, 66], parietal alpha oscillations have typically been considered as closely related to the visual system [28].

The involvement of the parietal cortex in the somatosensory detection task went unnoticed in the first analysis of the data that have been reanalyzed in the present paper [33]. In our new analysis, we used the SGPD to more effectively explore the data, looking for interesting spatiotemporal effects. This led to the identification of a suppression of alpha amplitude that originates from the parietal cortex and then propagates to the somatosensory regions. This effect turned out to be statistically robust when tested in a second independent dataset that was collected in the same experiment. The results suggest a hierarchical organization of the reconfiguration of alpha amplitude following an attentional cue. In particular, the initial reduction of parietal alpha amplitude could reflect the activation of a supramodal attentional network that paves the way for later sensorimotor-specific cortical reconfiguration.

While we mainly restricted our attention to the analysis of alpha oscillations, we believe that the GP-based decomposition can be useful for the study of other neural oscillations as well as non-rhythmic components. Several experimental tasks are related to effects in multiple dynamic components. For example, perception of naturalistic videos induces modulations in several frequency bands [67]. Studying the interplay between these differential modulations requires an appropriate decomposition of the measured signals that can be effectively performed using GP-based decomposition.

### Computational efficiency

The time complexity of SGPD is separately cubic in the number of time points *M* and and in the number of sensors *N*. In fact, the method involves the inversion of both the spatial covariance matrix (*N* × *N*) and the temporal covariance matrix (*M* × *M*) (see Eqs 37 and 38 in the Methods). For MEG or EEG applications, the inversion of the spatial covariance matrix is not problematic as the number of sensors is rarely much larger than 300. In several neuroscience applications, the data are analyzed in short trials and the cubic complexity in the number of time points (tipically ranging from 300 to 1000) is not particularly problematic either. However, this complexity could be prohibitive when analyzing long continuous signals. Fortunately, several approximate and exact methods have been introduced for reducing the complexity of GP regression to quadratic or even linear in the number of time points (see for example [46, 68, 69]). For example, the GP regression can be transformed into an infinite-dimensional version of the Kalman smoother that has linear complexity in the number of time points [46]. Finally, in terms of memory requirements, working in the spherical harmonic domain is convenient as the number of required harmonics is often an order of magnitude smaller than the number of source points in the cortical mesh.

### Conclusions

Our dynamic decomposition method starts from a precise mathematical model of the dynamics of the neural fields. The formalism of GP regression allows translation of linear stochastic dynamics into a well-defined Bayesian prior distribution. In this way, the method establishes a connection between mathematical modeling and data analysis of neural phenomena. On the one hand, the experimentalist and the data-analyst can benefit from the method as it allows to isolate the dynamic components of interest from the interfering noise. These components are interpretable and visualizable, and their study can lead to the identification of new temporal and spatiotemporal neural phenomena that are relevant for human cognition. On the other hand, the theorist can use this formalism for obtaining a probabilistic formulation of dynamical models, thereby relating them to the experimental data.

## Materials and methods

In this section we will explain the mathematical underpinnings of the GP-based decomposition. Following the lines of the Results section, the exposition begins from the connection between SDEs and Gaussian processes and continues with the exposition of the temporal and spatiotemporal GP-based decomposition. In order to improve the readability and to not overshadow the main ideas, we left some technical derivations to the appendices.

### From SDEs to GPs

At the core of our method is the connection between Gaussian processes and SDEs. This connection leads to the definition of the covariance functions of the dynamic components that will be used for determining the prior of the GP regression. In the Results section, we introduced the SDE (Eq (1))

$$\frac{d^{2}}{dt^{2}}\varphi \left(t\right)+b\frac{d}{dt}\varphi \left(t\right)=-\omega_{0}^{2}\varphi \left(t\right)+w\left(t\right)$$

to model an oscillatory signal. In fact, this SDE can be interpreted as a damped harmonic oscillator when $b<\sqrt{2\omega_{0}^{2}}$. As initial conditions, we set $\varphi \left(-\infty \right)=\frac{d\varphi }{dt}\left(-\infty \right)=0$. This choice implies that the (deterministic) effects of the initial conditions are negligible. Given these initial conditions, the solution of Eq (1) is fully specified by the random input *w*(*t*) that follows a temporally uncorrelated normal distribution. Since the equation is linear, the solution, given a particular instantiation of *w*(*t*), can be obtained by convolving *w*(*t*) with the impulse response function of the SDE (see the supporting information for more details):

$$\begin{array}{c} \varphi \left(t\right)=\int_{-\infty }^{\infty }G_{\varphi }\left(t-s\right)w\left(s\right)ds. \end{array}$$ (7)

Intuitively, the impulse response function *G*_*φ*_(*t*) determines the response of the system to a localized unit-amplitude input. Consequently, Eq (7) states that the process *φ*(*t*) is generated by the infinite superposition of responses to *w*(*t*) at every time point. This proves that the resulting stochastic process *φ*(*t*) is Gaussian, since it is a linear mixture of Gaussian random variables.

The impulse response function of Eq (1) is

$$\begin{array}{c} G_{\varphi }\left(t\right)=\vartheta \left(t\right)e^{-b/2t}\sin\omega t, \end{array}$$ (8)

where *ϑ*(*t*) is a function equal to zero for *t* < 0 and 1 otherwise. This function assures that the response cannot precede the input impulse. From this formula, we see that the system responds to an impulse by oscillating at frequency $\omega =\sqrt{\omega_{0}^{2}-1/4b^{2}}$ and with an amplitude that decays exponentially with time scale *b*/2. The covariance function of the process *φ*(*t*) can be determined from its impulse response function and is given by

$$\begin{array}{c} k_{\varphi }\left(t_{i},t_{j}\right)=k_{\varphi }\left(\tau \right)=\frac{\sigma_{\varphi }^{2}}{2b}e^{-b/2|\tau |}\left(\cos\omega \tau +\frac{b}{\omega }\sin\omega \left|\tau \right|\right). \end{array}$$ (9)

where *τ* denotes the time difference *t*_*i*_ − *t*_*j*_. In the case of the second order integrator, the parameter *ω*_0_ is smaller than *b*/2 and the system is overdamped. In this case, the response to an impulse is not oscillatory, the response initially rises and then decays to zero with time scale *b*/2. This behavior is determined by the impulse response function

$$\begin{array}{c} G_{\chi }\left(t\right)=\vartheta \left(t\right)e^{-b/2t}\sinh zt \end{array}$$ (10)

in which *z* is equal to $\sqrt{1/4b^{2}-\omega_{0}^{2}}$. The covariance function is given by

$$\begin{array}{c} k_{\chi }\left(\tau \right)=\frac{\sigma_{\chi }^{2}}{2b}e^{-b/2|\tau |}\left(\cosh z\tau +\frac{b}{z}\sinh z\left|\tau \right|\right). \end{array}$$ (11)

Finally, the first order integrator (Eq (2))

$$\;\frac{d}{dt}\psi \left(t\right)-c\psi \left(t\right)+w\left(t\right)$$

has a discontinuous impulse response function that decays exponentially:

$$\begin{array}{c} G_{\psi }\left(t\right)=\vartheta \left(t\right)e^{-ct}\;. \end{array}$$ (12)

The discontinuity of the impulse response at *t* = 0 implies that the process is not differentiable as it reacts very abruptly to the external input. The covariance function of this process is given by:

$$\begin{array}{c} k_{\psi }\left(\tau \right)=\frac{\sigma_{\psi }^{2}}{2c}e^{-c|\tau |}\;. \end{array}$$ (13)

#### Covariance function for the residuals

The stochastic differential equations are meant to capture the most important (linear) qualitative features of the neural signal. Nevertheless, the real underlying neural dynamics are much more complex than can be captured by any simple model. Empirically, we found that the residuals of our model have short-lived temporal correlations. We decided to account for these correlations by introducing a residuals process *ξ*(*t*) with covariance function

$$\begin{array}{c} k_{\xi }\left(\tau \right)=\sigma_{\xi }^{2}e^{-\frac{\tau^{2}}{2\delta^{2}}} \end{array}$$ (14)

in which the small time constant *δ* is the signal’s characteristic time scale and *σ*_*ξ*_ is its standard deviation. This covariance function is commonly called the squared exponential and is one of the most used in the machine learning literature [10]. As *k*_*ξ*_(*τ*) decays to zero much faster than our SDE-derived covariance functions for *τ* tending to ∞, this covariance function is appropriate for modeling short-lived temporal correlations.

### Analysing neural signals using Gaussian process regression

In this section, we show how to estimate the value of a dynamic component such as *φ*(*t*) in the set of sample points $t_{1},\ldots ,t_{N}$ using GP regression. To this end, it is convenient to collect all the components other than *φ*(*t*) in a total residuals process *ζ*(*t*) = *χ*(*t*) + *ψ*(*t*) + *ξ*(*t*). In fact, in this context, they jointly have the role of interfering noise. The vector of data points ***y*** is assumed to be a sum of the signal of interest and the noise:

$$\begin{array}{c} y_{j}=\varphi \left(t_{j}\right)+\zeta \left(t_{j}\right)\;. \end{array}$$ (15)

In order to estimate the values of *φ*(*t*) using Bayes’ theorem we need to specify a prior distribution over the space of continuous-time signals. In the previous sections, we saw how to construct such probability distributions from linear SDEs. In particular, we found that those distributions were GPs with covariance functions that can be analytically obtained from the impulse response function of the SDEs. These prior distributions can be summarized in the following way:

$$\begin{array}{cc} \varphi (t) & ∼GP(0,k_{\varphi }\left(t_{1},t_{2}\right))\\ \zeta (t) & ∼GP(0,k_{\zeta }\left(t_{1},t_{2}\right)) \end{array}$$ (16)

where the symbol ∼ indicates that the random variable on the left-hand side follows the distribution on the right-hand side and *GP*(*μ*(*t*), *k*(*t*_1_, *t*_2_)) denotes a GP with mean function *μ*(*t*) and covariance function *k*(*t*_1_, *t*_2_). Note that, in this functional notation, expressions such as *μ*(*t*) and *k*(*t*_1_, *t*_2_) denote whole functions rather than just the values of these functions at specific time points.

We will now derive the marginal expectation of *φ*(*t*) under the posterior distribution. Since we are interested in the values of *φ*(*t*) at sample points *t*_1_,…, *t*_*N*_, it is convenient to introduce the vector ***φ*** defined by the entries *φ*_*j*_ = *φ*(*t*_*j*_). Any marginal distribution of a GP for a finite set of sample points is a multivariate Gaussian whose covariance matrix is obtained by evaluating the covariance function at every pair of time points:

$$\begin{array}{c} {\left[K_{\varphi }\right]}_{ij}=k_{\varphi }\left(t_{i},t_{j}\right). \end{array}$$ (17)

Using Bayes’ theorem and integrating out the total residual *ζ*(*t*), we can now write the marginal posterior of ***φ*** as

$$\begin{array}{c} p\left(\varphi |y\right)\propto \int p\left(y|\varphi ,\zeta \right)p\left(\zeta \right)d\zeta \;p\left(\varphi \right)=N\left(y|\varphi ,K_{\zeta }\right)N\left(\varphi |0,K_{\varphi }\right) \end{array}$$ (18)

in which *K*_*ζ*_ is the temporal covariance matrix of *ζ*(*t*). As a product of two Gaussian densities, the posterior density is a Gaussian distribution itself. The parameters of the posterior can be found by writing the prior and the likelihood in canonical form. From this form, it is easy to show that the posterior marginal expectation is given by the vector ***m***_*φ*|*y*_ (see [10] for more details about this derivation):

$$\begin{array}{c} m_{\varphi |y}=K_{\varphi }{\left(K_{\varphi }+K_{\zeta }\right)}^{-1}y. \end{array}$$ (19)

Furthermore, if we assume that *χ*(*t*), *ψ*(*t*) and *ξ*(*t*) are independent, the noise covariance matrix reduces to

$$\begin{array}{c} K_{\zeta }=K_{\chi }+K_{\psi }+K_{\xi }. \end{array}$$ (20)

### GP analysis of spatiotemporal signals

In the following, we show how to generalize GP-based decomposition to the spatiotemporal setting. This requires the construction of a source model and the definition of an appropriate prior covariance between cortical locations. In fact, the problem of localizing brain activity from MEG or EEG sensors becomes solvable once we introduce prior spatial correlations by defining a spatial covariance *s*(***x***_*i*_, ***x***_*j*_) between every pair of cortical locations ***x***_*i*_ and ***x***_*j*_. In this paper, we construct *s*(***x***_*i*_, ***x***_*j*_) by discounting high spatial frequencies in the spherical harmonics domain, thereby limiting our reconstruction to spatial scales that can be reliably estimated from the sensor measurements. However, prior to the definition of the covariance function, we need to specify a model of the geometry of the head and the brain cortex.

#### The source model

In order to define a source model, we construct a triangular mesh of the cortex from a structural MRI scan using Freesurfer [23]. The cortical boundary is morphed into a spherical hull in a way that maximally preserves the intrinsic geometry of the cortex. This allows to parameterize the surface *C* using the spherical coordinates *α* and *θ*, respectively azimuth and elevation. For notational simplicity, we collect the spherical coordinates into the coordinate pair ***x*** = (*α*, *θ*) that refers to a spatial location in the cortex. Furthermore, we denote the finite set of *M* points in the mesh as $X=\{x_{1},\ldots ,x_{M}\}$.

We define our source model as a vector field of current dipoles on the cortical surface. We first consider GP source reconstruction of the total neural activity $\vec{\rho }\left(x,t\right)$, without differentiating between spatiotemporal dynamic components such as $\vec{\varphi }\left(x,t\right)$, $\vec{\chi }\left(x,t\right)$ and $\vec{\psi }\left(x,t\right)$. The vector field $\vec{\rho }\left(x,t\right)$ is characterized by the three Cartesian coordinates *ρ*_1_(*x*, *t*), *ρ*_2_(*x*, *t*), and *ρ*_3_(*x*, *t*). In all the analyses contained in this paper, we estimate the full vector field. However, since we do not assume any prior correlations between the dipole coordinates, in the following we will simplify the notation by describing the source decomposition method for a dipole field $\rho \left(x,t\right)\vec{v}\left(x\right)$, where the unit-length vector field $\vec{v}\left(x\right)$ of dipole orientations is assumed to be known. The supporting information contains an explanation of how to adapt all the formulas to the vector-valued case using matrices with a block diagonal form.

#### Spatial Gaussian processes source reconstruction in the spherical harmonics domain

The linearity of the electromagnetic field allows to model the spatiotemporal data matrix *Y* as the result of a linear operator acting on the neural activity *ρ*(*x*, *t*) [31]:

$$\begin{array}{c} Y_{ij}=\int_{C}L_{i}\left(x\right)\rho \left(x,t_{j}\right)dx\;, \end{array}$$ (21)

in which the component $L_{i}\left(x\right)$ describes the effect of a source located at ***x*** on the *i*-th sensor. Note that $L_{i}\left(x\right)$ implicitly depends on the orientation $\vec{v}\left(x\right)$ since different dipole orientations generate different sensor measurements. We refer to $L_{i}\left(x\right)$ as the forward model relative to the *i*-th sensor, note that this is a function of the spatial location on the cortical surface.

**Fig 9:**
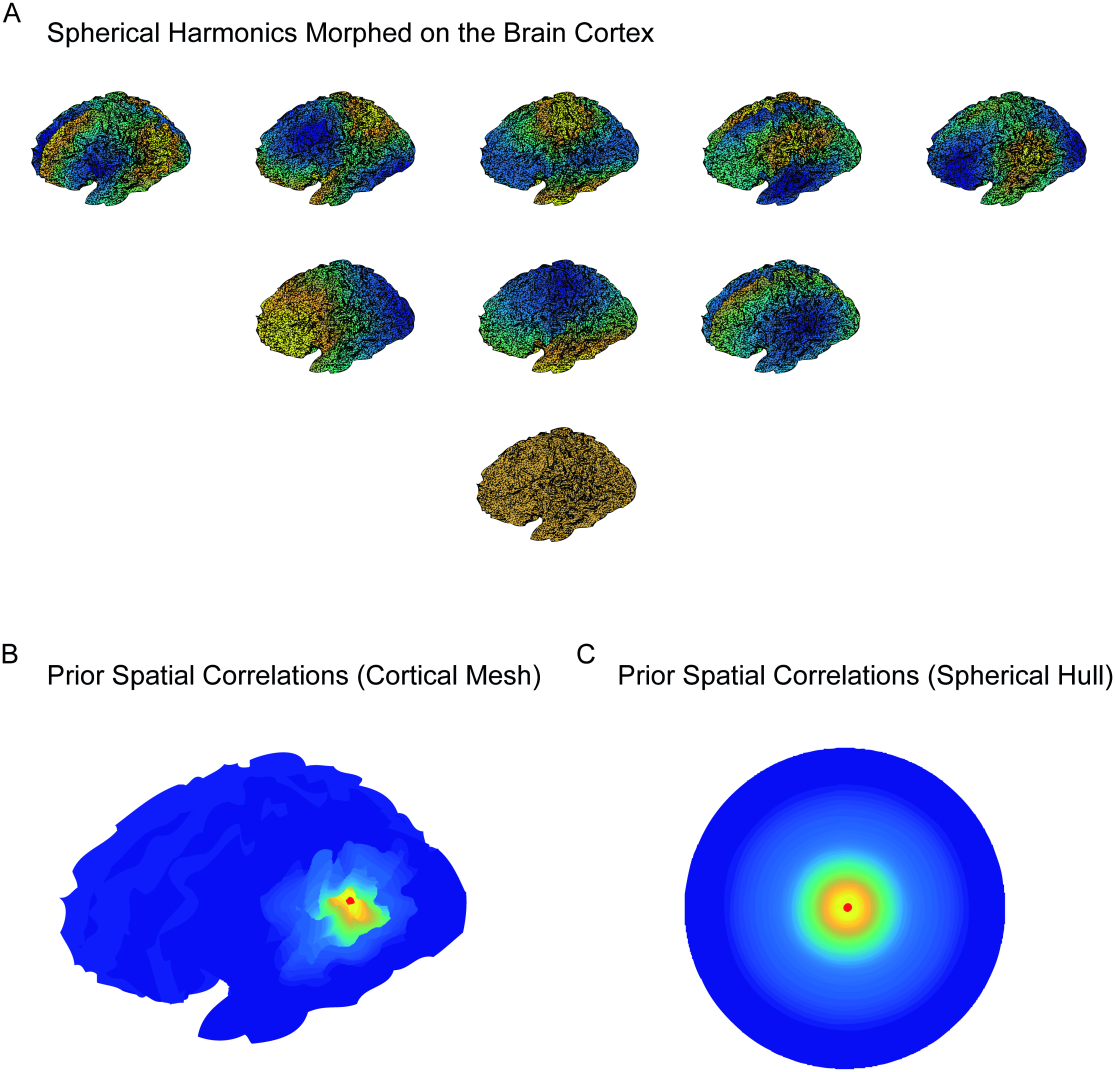
Spherical harmonics and covariance functions. Visualization of the spherical harmonics morphed onto the cortex and the resulting spatial correlation structure. A) Example of spherical harmonics on the brain cortex for frequency numbers from 0 to 2. For each frequency number *l* there are 2*l*+1 harmonics with “phase” number *m* ranging from −*l* to *l*. As clear from the picture, the spatial frequency increases as a function of the frequency number. In all our analyses we truncated the harmonic expansion after the 11th frequency number. B,C) Prior correlation structure induced by Eq (24). Panel B shows the prior correlations on the cortical surface from a cortical point identified by a red dot. Panel C shows the same function on the spherical hull. The spatial correlations are determined by the frequency discount function *f*(*l*); here we used the same smoothing parameters as all analyses in the paper: *k* = 2 and *υ* = 3.

In this section, we ignore the prior temporal correlations induced by the temporal covariance functions, i.e. we implicitly assume a prior for *ρ*(***x***, *t*) that is temporally white. In a GP regression setting, the spatial smoothing can be implemented by using a spatially homogeneous covariance function, i.e. a covariance function that only depends on the cortical distance between the sources. To define this covariance function, we make use of the so-called spherical Fourier transform. Whereas the ordinary Fourier transform decomposes signals into sinusoidal waves, the spherical Fourier transform decomposes spatial configurations defined over a sphere into the spherical harmonics $H_{l}^{m}\left(x\right)$. These basis functions are characterized by a spatial frequency number *l* and a “spatial phase” number *m*. Fig 9A shows the spherical harmonics corresponding to the first three spatial frequencies morphed on the cortical surface. For notational convenience, we assign an arbitrary linear indexing to each (*l*, *m*) couple that henceforth will be denoted as (*l*_*k*_, *m*_*k*_). It is convenient to represent the neural activity *ρ*(***x***, *t*) in the spherical harmonics domain. Specifically, we will use the symbol $\tilde{\rho }\left(l_{k},m_{k};t\right)$ to denote the Fourier coefficient of the spherical harmonic indexed by (*l*_*k*_, *m*_*k*_) (see the supporting information).

We assume that the spherical Fourier coefficients $\tilde{\rho }\left(l_{k},m_{k};t\right)$ are independent Gaussian random variables. Under this assumption, we just need to define the prior variance of the coefficients $\tilde{\rho }\left(l_{k},m_{k};t\right)$. Since we aim to reduce the effect of noise with high spatial frequencies, we define these prior variances using a frequency damping function *f*(*l*_*k*_) that monotonically decreases as a function of the spatial frequency number *l*_*k*_. This effectively discounts high spatial frequencies and therefore can be seen as a spherical low-pass filter. The variance of the spherical Fourier coefficients is given by the following variance function

$$\begin{array}{c} \tilde{s}\left(l_{k},m_{k};t\right)=f\left(l_{k}\right), \end{array}$$ (22)

where, as damping function, we use a spherical version of the truncated Butterworth low-pass filter:

$$\begin{array}{c} f\left(l_{k}\right)=\left\{\begin{array}{cc} {\left(1+{\left(\frac{l_{k}}{\upsilon }\right)}^{2k}\right)}^{-1/2} & \text{for}\;l_{k}\leq L\\ 0 & \text{for}\;l_{k}>L \end{array}\right. \end{array}$$ (23)

with smoothing parameter *υ*, order *k*, and cut-off frequency *L*. This filter has been shown to have good properties in the spatial domain [70]. Note that, under the covariance function defined by Eqs (22) and (23), the spherical Fourier coefficients with frequency number larger than *L* have zero variance and are therefore irrelevant. Although the analysis is carried out in the spherical harmonics domain, it is informative to be able to visualize the covariance function in the spatial domain. By applying the inverse spherical Fourier transform, the function *s*(***x***_*i*_, ***x***_*j*_) can be explicitly obtained as follows:

$$\begin{array}{c} s\left(x_{i},x_{j}\right)=\sum_{l,m}H_{l}^{m}\left(x_{i}\right)H_{l}^{m}\left(x_{j}\right)f\left(l\right)\;. \end{array}$$ (24)

Fig 9B and 9C show the correlations induced by our spatial covariance function.

In order to formulate the spatial GP regression in the spherical harmonics domain, we rewrite the integral in Eq (21) using the inverse spherical Fourier transform (see the supporting information) and interchanging the order of summation and integration:

$$\begin{array}{c} Y_{ij}=\int_{C}L_{i}\left(x\right)\left(\sum_{k}\tilde{\rho }\left(l_{k},m_{k};t_{j}\right)H_{l_{k}}^{m_{k}}\left(x\right)\right)dx=\sum_{k}{\tilde{L}}_{i}\left(l_{k},m_{k}\right)\tilde{\rho }\left(l_{k},m_{k};t_{j}\right), \end{array}$$ (25)

where

$$\begin{array}{c} {\tilde{L}}_{i}\left(l_{k},m_{k}\right)=\int_{C}L_{i}\left(x\right)H_{l_{k}}^{m_{k}}\left(x\right)dx \end{array}$$ (26)

is the spherical Fourier transform of $L_{i}\left(x\right)$. Therefore, the spherical Fourier transform converts the forward model (which is a function of the cortical location) from the spatial to the spherical harmonics domain. We can simplify Eq (25) by organizing the spherical Fourier coefficients $\tilde{\rho }\left(l_{k},m_{k};t_{j}\right)$ in the matrix $\tilde{R}$, whose element ${\tilde{R}}_{kj}$ is $\tilde{\rho }\left(l_{k},m_{k};t_{j}\right)$. Analogously, the spherical Fourier transform of the forward model can be arranged in a matrix Λ with elements $\Lambda_{ik}={\tilde{L}}_{i}\left(l_{k},m_{k}\right)$. Using this notation, we can write the observation model for the spatiotemporal data matrix *Y* in a compact way:

$$\begin{array}{c} Y=\Lambda \tilde{R}+\xi \;, \end{array}$$ (27)

where ***ξ*** are Gaussian residuals with spatial covariance matrix Σ.

We can now combine this observation model with the spherical harmonics domain spatial GP prior, as determined by the variance function given by Eq (22), and from this we obtain the posterior of the neural activity $\tilde{R}$ given the measured signal *Y*. Because the spatial process is Gaussian, the prior distribution of the spherical Fourier coefficients is normal and, because we assumed that the spherical Fourier coefficients are independent, their covariance matrix *D* is diagonal with entries specified by the variance function *D*_*kk*_ = *f*(*l*_*k*_) (see Eq (22)). Alltogether, the prior and the observation model specify a Gaussian linear regression. The posterior expectation of the regression coeffcients $\tilde{R}$ can be shown to be [15]:

$$\begin{array}{c} M_{\tilde{R}|Y}={D\Lambda }^{T}{{(\Lambda D\Lambda }^{T}+\Sigma )}^{-1}Y. \end{array}$$ (28)

In this formula, Λ*D*Λ^*T*^ is the sensor level covariance matrix induced by the spatially smooth brain activity and Σ is the residual covariance matrix of the sensors. This expression can be recast in terms of the original cortical locations X using the inverse spherical Fourier transform. In matrix form, this can be written as

$$\begin{array}{c} M_{R|Y}=HM_{\tilde{\rho }|Y}, \end{array}$$ (29)

where the matrix *H* is obtained by evaluating the spherical harmonics at the discrete spatial grid-points X:

$$\begin{array}{c} H_{lk}=H_{l_{k}}^{m_{k}}\left(x_{l}\right)\;. \end{array}$$ (30)

This formula gives the Harmony source reconstruction solution as presented in [16]. We can reformulate this expression by introducing the Harmony spatial filter

$$\begin{array}{c} P={HD\Lambda }^{T}{{(\Lambda D\Lambda }^{T}+\Sigma )}^{-1}. \end{array}$$ (31)

Using this matrix, the posterior expectation of the neural activity at the cortical locations X can be written as follows:

$$\begin{array}{c} M_{R|Y}=PY. \end{array}$$ (32)

#### Spatiotemporal GP-based decomposition

The temporal and spatial GP regression can be combined by assigning a temporal covariance function to each spherical Fourier coefficient. In other words, we model the time series of each coefficient as an independent temporal Gaussian process. These processes have the same prior temporal correlation structure as specified in our additive temporal model. However, as in the spatial model, their prior variance is discounted as a function of the spatial frequency *l*_*k*_. Using functional notation, this can be written as follows:

$$\begin{array}{c} \tilde{\rho }\left(l,m;t\right)∼GP\left(0,f\left(l\right)k_{\rho }\left(t_{1},t_{2}\right)\right). \end{array}$$ (33)

Considering the prior distributions of the processes $\tilde{\rho }\left(l,m;t\right)$ at the sample points, the matrix-valued random variable $\tilde{R}$, when vectorized, follows a multivariate Gaussian distribution with covariance matrix *K*_*ρ*_ ⊗ *D*, where ⊗ denotes the Kronecker product (see the supporting information). This Kronecker product form follows from the fact that the covariance function of $\tilde{\rho }\left(l,m;t\right)$ is the product of a spatial and a temporal part. Multivariate Gaussian distributions with this Kronecker structure can be more compactly reformulated as a matrix normal distribution (see [71]):

$$\begin{array}{c} \tilde{R}∼MN\left(0,D,K_{\rho }\right)\;, \end{array}$$ (34)

where the matrix parameters *D* and *K*_*ρ*_ determine the covariance structure across, respectively, the spherical harmonics and time.

We define a spatiotemporal observation model in which the residuals have a spatiotemporal covariance structure of the form *K*_*ξ*_ ⊗ (Λ*D*Λ^*T*^). This implies that the spatial covariance matrix of the residuals (previously denoted as Σ) has the form Λ*D*Λ^*T*^. Thus, it is assumed that the residuals have the same spatial covariance as the brain activity of interest (see Eq (28) but a different temporal covariance. Hence, *ξ*(***x***, *t*) should be interpreted as brain noise [72]. This assumption greatly simplifies the derivation of the posterior distribution. Under this observation model, the probability distribution of the spatiotemporal data matrix can be written as follows:

$$\begin{array}{c} Y∼MN(\Lambda \tilde{R},\Lambda D\Lambda^{T},K_{\xi })\;. \end{array}$$ (35)

The posterior expectation for this model can be obtained using the properties of Kronecker product matrices. This derivation is slightly technical and is reported in the supporting information. In this derivation, to enhance numerical stability, we introduce a Tikhonov regularization parameter *λ*. This allows us to deal with the fact that the matrix Λ*D*Λ^*T*^ (which must be inverted), is usually close to singular for an MEG or EEG forward model. The resulting posterior expectation is the following:

$$\begin{array}{c} M_{\tilde{R}|Y}={D\Lambda }^{T}{{(\Lambda D\Lambda }^{T}+\lambda I)}^{-1}Y{\left(K_{\rho }+K_{\xi }\right)}^{-1}K_{\rho }\;. \end{array}$$ (36)

Besides regularizing the matrix inversion, the *λI* term contributes to filtering out the spatially non-structured observation noise. This is consistent with the fact that the regularization matrix replaces the noise spatial covariance matrix in Eq (28) and, being diagonal, corresponds to spatially white noise. In the spatial domain, Eq (36) becomes:

$$\begin{array}{c} M_{R|Y}=PY{\left(K_{\rho }+K_{\xi }\right)}^{-1}K_{\rho }\;. \end{array}$$ (37)

Therefore, the spatiotemporal expectation is obtained by applying the Harmony spatial filter (with Σ = *λI*) to the expectation of the temporal model given by Eq (19). We can now apply this to the situation in which we want to estimate some component of interest, such as *φ*(***x***, *t*), in the presence of other components *ζ*(***x***, *t*) = *χ*(***x***, *t*) + *ψ*(***x***, *t*) + *ξ*(***x***, *t*). In analogy with Eq (19), the marginal expectation of the spatiotemporal component *φ*(***x***, *t*) is given by

$$\begin{array}{c} M_{\Phi |Y}=PY{\left(K_{\varphi }+K_{\zeta }\right)}^{-1}K_{\varphi }\;, \end{array}$$ (38)

where *K*_*ζ*_ is the temporal covariance matrix of *ζ*(***x***, *t*) = *χ*(***x***, *t*) + *ψ*(***x***, *t*) + *ξ*(***x***, *t*). This formula allows to individually reconstruct the dynamic components.

### Estimating the model parameters

We estimate the parameters of the covariance functions from all the data of each participant using an empirical Bayes method. This produces a prior distribution that is both informed by the participant-specific signal dynamics and flexible enough to account for the variability across different epochs. Specifically, given *K* trials, the parameters are estimated from the empirical autocovariance matrix *S* of the total measured time series:

$$\begin{array}{c} S=\sum_{k=1}^{K}Y_{k}Y_{k}^{T} \end{array}$$ (39)

where *Y*_*k*_ denotes the demeaned (mean-subtracted) spatiotemporal data matrix of an experimental trial *k*. For notational convenience, we organize all the parameters of the model covariance function in the vector *ϑ*. Furthermore, we make the dependence on the parameters explicit by denoting the total covariance function of the total additive model as

$$\begin{array}{c} k_{\rho }\left(t,t^{\prime };\vartheta \right)=k_{\varphi }\left(t,t^{\prime };\vartheta \right)+k_{\chi }\left(t,t^{\prime };\vartheta \right)+k_{\psi }\left(t,t^{\prime };\vartheta \right)+k_{\xi }\left(t,t^{\prime };\vartheta \right). \end{array}$$ (40)

As the objective function to be minimized, we use the sum of the squared deviations of the measured time series’ auto-covariance from the covariance function of our model:

$$\begin{array}{c} C\left(\vartheta \right)=\sum_{i,j}{\left(S_{ij}-k_{\rho }\left(t_{i},t_{j};\vartheta \right)\right)}^{2} \end{array}$$ (41)

This objective function is, in general, multimodal and requires the use of a robust optimization technique. Gradient-based methods can be unstable since they can easily lead to sub-optimal local-minima. For that reason we used a gradient-free simulated annealing strategy. The details of the simulated annealing algorithm are described in [24]. As proposal distribution we used

$$\begin{array}{c} p\left(\vartheta_{j}^{\left(k+1\right)}\right)=t\left(\vartheta_{j}^{\left(k+1\right)}|\vartheta_{j}^{\left(k\right)},\gamma_{j},1\right)\;, \end{array}$$ (42)

where *t*(*x*|*a*, *b*, *c*) denotes a univariate Student’s t-distribution over *x* with mean *a*, scale *b* and *c* degrees of freedom. We chose this distribution because the samples can span several order of magnitudes, thereby allowing both a quick convergence to the low cost region and an effective fine tuning at the final stages. We used the following annealing schedule:

$$\begin{array}{c} T(n+1)=0.8\cdot T(n)\;, \end{array}$$ (43)

where *T*(0) was initialized at 10 and the algorithm stopped when the temperature was smaller than 10^−8^.

We estimated all the temporal parameters of the model. Specifically, the estimated parameters were the following: (a) the alpha frequency $\omega =\sqrt{\omega_{0}^{2}-1/4b^{2}}$, phase decay *β*_*φ*_ = 1/2*b*_*φ*_, and amplitude $A_{\varphi }=\sigma_{\varphi }/\sqrt{2b_{\varphi }}$, (b) the second order integrator parameters *z*, *β*_*χ*_ = 1/2*b*_*χ*_, and its amplitude $A_{\chi }=\sigma_{\chi }/\sqrt{2b_{\chi }}$, (c) the first order integrator decay constant *c* and its amplitude $A_{\psi }=\sigma_{\psi }/\sqrt{2b_{\psi }}$, and (d) the residual’s time scale *δ*, and standard deviation *σ*_*ξ*_. The parameters were initialized at plausible values (e.g. 10 Hz for the oscillator frequency) and were constrained to stay within realistic intervals (6–15 Hz for alpha frequency, positive for *β*_*φ*_, *β*_*χ*_, *c*, *δ* and all the amplitudes).

### Details of the simulation studies

In this subsection we describe our three simulation studies in detail.

#### Simulation study I: Recovering components from complex spatiotemporal neural signals

The oscillatory time series were generated as a non-Gaussian random process according to the following formula:

$$\begin{array}{c} y\left(t\right)=\sqrt{a^{2}\left(t\right)+1}\cos\left(\omega \left(t\right)t+\gamma \right)+\xi \left(t\right)+\psi \left(t\right)\;. \end{array}$$ (44)

The random initial phase *γ* in this formula was drawn from a uniform distribution and the functions *a*(*t*) and *ω*(*t*) are Gaussian processes with a squared exponential covariance function (see Eq (14)). The noise processes *ξ*(*t*) and *ψ*(*t*) were respectively an OU process (with relaxation coefficient equal to 10) and white noise. The source model was one-dimensional, composed of 9 different spatial locations from -2 mm to 2 mm in steps of 0.5 mm. The oscillatory processes had a Gaussian spatial profile with the center located at -0.5 mm for the low frequency oscillation and 0.5 mm for the high frequency oscillation. There were two independent sources of OU noise (with standard deviation equal to 0.6 each) with Gaussian spatial profiles with centers located at -1.5 mm and 1.5 mm and width equal to 1 mm. Finally, the data was corrupted by spatiotemporal white noise (with standard deviation equal to 0.1). We had four conditions: a) low spatial and low spectral overlap, b) low spatial and high spectral overlap, c) high spatial and low spectral overlap and d) high spatial and high spectral overlap. The peak frequency of the slow oscillation was 5 Hz for low spectral overlap conditions and 8 Hz for high spectral overlap conditions while the peak frequency of the fast oscillation was always 10 Hz. In the low and in the high spatial overlap conditions the widths of the spatial profiles of both oscillations were respectively 1 mm and 2 mm. For each condition, we generated 600 trials. The fast oscillation was reconstructed using SGPD, SSA, EMD, ICA and PCA. The spatial covariance matrix for the SGPD was obtained by discounting the spatial frequencies using the discount function in Eq 23. The parameters were *l* = 0.2 and *k* = 5. The additive temporal covariance function of the SGPD had two oscillatory components, a first order integrator component and a squared exponential residual component. All the parameters of the temporal covariance function were inferred from the data using the least squares fit. Performance of each method was evaluated by computing the correlation between the true oscillatory signal (ground truth) and the recovered fast oscillatory component. Since the components obtained from SSA, EMD, ICA and PCA are unlabeled, for these methods we selected the component that maximized the correlation.

#### Simulation study II: Estimating modulations in oscillatory amplitude

The study consisted of two simulated experimental conditions that differed only with respect to their mean oscillatory amplitude. In this simulation the signals were purely temporal, as we did not model the spatial extent of the sources. In each trial a single oscillatory time series was generated using Eq 44. The mean of its angular frequency *ω*(*t*) was equal to 2*π* ⋅ 10 (the typical frequency of alpha oscillations) for both experimental conditions. In the low-amplitude condition the mean oscillatory amplitude was equal to 1. This oscillatory time series was corrupted by an OU process (amplitude equal to 0.55; relaxation coefficient equal to 10) and white noise (amplitude equal to 0.55). The simulation design involved 16 levels characterized by amplitude differences ranging from 15% to 60%. For each level, we generated 150,000 trials per experimental condition, giving us very reliable estimates of the effect size. The trials were 2 s long. We used the temporal GP-based decomposition to extract the oscillatory component from the simulated time series. The effect sizes were quantified as the between-condition differences between the trial-averaged amplitudes divided by the across-trials standard deviation of the amplitudes. We compared the sensitivity of the GP-based decomposition with non-parametric spectral estimation using DPSS multitaper spectral analysis as described in [30]. For every trial, the mean oscillatory amplitude was obtained by averaging over the amplitude estimates for the orthogonal tapers. In this method, the number of tapers is a free parameter that determines the degree of spectral smoothing. For each cell of the simulation design we chose the number of tapers that maximized the effect size. This selection procedure is biased in favor of the multitaper method since it tends to overfit the data and therefore produces larger effect sizes.

#### Simulation study III: Localizing the source of an oscillatory amplitude modulation

A template cortical surface mesh was created using Freesurfer [23], down-sampled using the MNE toolbox [73], and aligned to a template MEG sensor configuration. We ran 500 trials, each involving two conditions that differed only with respect to the oscillatory amplitude of one cortical location. Sources were generated at three locations in the brain: one in the right parieto-temporal, one in the right occipital and one in the left parietal cortex. For each trial and condition, we generated three time series with the same temporal structure as those generated in the previous simulation study. The three time series were localized in cortical mesh with a spatial profile that is proportional to a Fisher-von Mises distribution. These spatial profiles can model a localized patch of activity. The dipole orientation was set to be orthogonal to the mesh surface. While all patches of activity contained the oscillatory component, only one patch involved an amplitude modulation between the two experimental conditions, and this was set at 20%. The activity was projected to the MEG sensors using a forward model obtained from a realistic head model [31]. The effect was computed for each cortical vertex as the difference in average oscillatory amplitude between the two conditions.

The oscillatory signal was first reconstructed at each cortical vertex using the spatiotemporal GP-based decomposition. Next, as in the simulation study for the single sensor, the GP estimate of average oscillatory amplitude was obtained as the standard deviation of the estimate of the oscillatory component. We compared the spatiotemporal GP-based decomposition with the Harmony source reconstruction of the estimated cross-spectral density matrix. Using the DPSS multitaper spectral analysis, we first estimated the sensor-level cross-spectral density matrix *F*. Next, we projected this matrix to the source level by sandwiching it between the Harmony spatial filters (see Eq (29)): *F*_*H*_ = *PFP*^*T*^. The source level amplitude is obtained by taking the square root of the diagonal elements of *F*_*H*_. The spectral smoothing was kept fixed at 0.6 Hz since we found this value to be optimal given the simulation parameters.

### Details of the application to an MEG study on anticipatory spatial attention

#### Participants and data collection

We tested the spatiotemporal GP source reconstruction method on a cued tactile detection experiment in which the magneto-encephalogram (MEG) was recorded [26]. The study was conducted in accordance with the Declaration of Helsinki and approved by the local ethics committee (CMO Regio Arnhem-Nijmegen). Informed written consent was obtained from all participants. Fourteen healthy participants (5 male; 22–49 yr) participated in the study. The MEG system (CTF MEG; MISL, Coquitlam, British Columbia, Canada) had 273 axial gradiometers and was located in a magnetically shielded room. The head position was determined by localization coils fixed to anatomic landmarks (nasion and ears). The data were low-pass filtered (300-Hz cutoff), digitized at 1,200 Hz and stored for offline analysis.

#### Experimental design

The experiment was a tactile detection task in which the location and timing of the targets were either cued or not. A short auditory stimulus (50 ms, white noise) was presented together with an electrotactile stimulus (0.5-ms electric pulse close to threshold intensity) in half of the trials. In the other half the auditory stimulus was presented alone. Participants were asked to indicate if a tactile stimulus was presented. In one-third of the trials, an auditory cue (150 ms, pure tone) informed the participants about the timing and the location at which the tactile stimulus might occur. In particular, the target auditory signal was always presented 1.5 s after the cue. Two independent sessions were collected for each participant. More details can be found in [33].

#### MEG preprocessing

Third-order synthetic gradients were used to attenuate the environmental noise [74]. In addition, extra-cerebral physiological sources such as heartbeat and eye movements were detected using independent component analysis [11] and regressed out from the signal prior to the spatiotemporal GP-based decomposition.

#### Details of the GP spatiotemporal data analysis

We started the GP analysis by learning the parameters of the additive dynamical model for each individual participant using the simulated annealing method. To reduce the contribution of low-amplitude noise, we estimated this matrix from the first 50 principal components of the total empirical temporal cross-covariance matrix averaged over all sensors. A template cortical surface mesh was created using Freesurfer [23], downsampled using the MNE toolbox [73], and aligned to the MEG sensors using the measured head position. The Tikhonov regularization parameter *λ* was identified for each participant using leave-one-out cross-validation [32]. The spatial smoothing parameters *k* and *υ* were set to, respectively, 2 and 3. The spatiotemporal GP-based decomposition was applied to 1.8 s long segments, starting ten milliseconds before the presentation of the cue and ending ten milliseconds after the target stimulus. The alpha amplitude envelope *A*(*t*, *x*) was obtained for all cortical vertices and dipole directions by performing a Hilbert transform on the estimated alpha signal and taking the absolute value of the resulting analytic signal [61]. For each cortical location, the total amplitude was obtained by summing the amplitude envelopes for the three independent dipole directions *φ*_1_(***x***, *t*), *φ*_2_(***x***, *t*), and *φ*_3_(***x***, *t*). The individual topographic maps of the attention-induced alpha amplitude suppression were obtained by computing the mean amplitude difference between cued and non-cued trials, separately for each vertex and time point. These individual maps were then averaged across participants, again for each vertex and time point.

#### Statistical analysis

For each cortical vertex, the dynamic effect was quantified as the rate of change of the attention-induced alpha amplitude suppression as a function of elapsed time from cue onset. Specifically, we used linear regression to estimate the slope of the relation between attention-induced alpha amplitude suppression and time. We did this separately for every vertex. The cortical maps of regression coefficients were constructed from the first experimental session of every participant and then averaged across participants. This map was subsequently used as data-driven hypothesis which was tested using the data from the second session. As a test statistic, we used the dot product between the individual regression coefficients maps, computed from the second sessions, and the group-level map. Under the null hypothesis that the group-level map is not systematic (i.e., is driven by noise only), the expected value of this test statistic is zero. Therefore we tested this null hypothesis using a one-sample t-test.

## Supporting information

S1 Supporting Information S1 Appendix: Covariance functions defined by linear SDEs. Brief review of the technique used for obtaining the covariance function from the SDE. S2 Appendix: Spherical harmonics and spherical Fourier transform. Brief review of the spherical Fourier transform. S3 Appendix: Properties of the Kronecker product and GP regression with separable covariance matrices. Derivation of the spatiotemporal posterior expectation using Kronecker product matrices. S4 Appendix: Modeling vector-valued sources using block matrices. Generalization of the method to vector valued dipolar sources.
(PDF)
