# Supplementary material for: Dynamic decomposition of spatiotemporal neural signals
Source: PLoS Comput Biol. 2017 May 30;13(5):e1005540. doi: 10.1371/journal.pcbi.1005540 (PMC5469506; doi:10.1371/journal.pcbi.1005540)
Supplement: S1 Supporting Information — S1 Appendix: Covariance functions defined by linear SDEs. Brief review of the technique used for obtaining the covariance function from the SDE. S2 Appendix: Spherical harmonics and spherical Fourier transform. Brief review of the spherical Fourier transform. S3 Appendix: Properties of the Kronecker product and GP regression with separable covariance matrices. Derivation of the spatiotemporal posterior expectation using Kronecker product matrices. S4 Appendix: Modeling vector-valued sources using block matrices. Generalization of the method to vector valued dipolar sources. (PDF) [file pcbi.1005540.s001.pdf]

Dynamic decomposition of temporal and spatiotemporal neural signals

Luca Ambrogioni[1], Marcel van Gerven[1], Eric Maris[1],

1 Radboud University Donders Institute, Nijmegen, Netherlands

Supporting information

S1 Appendix: Covariance functions defined by linear SDEs

Consider a general linear SDE of the form

$$\sum_{k}^{K} c_k \frac{d^k \alpha(t)}{dt^k} = w(t) \tag{1}$$

where the coefficients c_k are chosen in a way to have stable solutions. An important tool for analyzing a linear differential equation is the impulse response function $G(t)$. This function is defined as the response of the system to a unit-amplitude impulse $\delta(t)$:

$$\sum_{k}^{K} c_k \frac{d^k G(t)}{dt^k} = \delta(t) \tag{2}$$

Using the impulse response function, a solution of the linear SDE driven by an arbitrary random input $w(t)$ can be written as follows:

$$\alpha(t) = \int_{-\infty}^{\infty} G(t-s) w(s) ds. \tag{3}$$

This means that the stochastic process $\alpha(t)$ is an infinite linear superposition of responses to the random uncorrelated input $w(s)$.

Using Eq. (3) we can derive the mean and covariance function of $\alpha(t)$. The mean function is defined as

$$m_\alpha(t) = \langle \alpha(t) \rangle, \tag{4}$$

where the triangular brackets $\langle \cdot \rangle$ denote the expectation with respect of the distribution of the random input $w(s)$. Using (3) in (4), we obtain:

$$m_\alpha(t) = \int_{-\infty}^{\infty} G(t-s) \langle w(s) \rangle ds = 0. \tag{5}$$

Here, we used the fact that the order of expectation and integration can be interchanged and that the expectation of the white noise process is equal to zero. Analogously, we can obtain the covariance function as follows:

$$k_\alpha(t,t') = \langle \alpha(t)\alpha(t') \rangle = \int_{-\infty}^{\infty} \int_{-\infty}^{\infty} G(t-s) G(t'-s') \langle w(s) w(s') \rangle ds ds'. \tag{6}$$

Since $w(s)$ is white, its covariance $\langle w(s)w(s')\rangle$ is given by the delta function $\sigma_\alpha^2\delta(s-s')$, where σ_α^2 is the variance of the random input. The integral over s' can be solved by using the translation property of the delta function:

$$\int_{-\infty}^{\infty} \delta(s-s')G(t'-s')ds' = G(t'-s). \tag{7}$$

Using this formula and introducing the new integration variable s^* equal to $t'-s$, the covariance function becomes

$$k_\alpha(t,t') = \sigma_\alpha^2 \int_{-\infty}^{\infty} G(t-t'+s^*)G(s^*)ds^*. \tag{8}$$

Since the covariance function depends on t and t' only though their difference $\tau = t-t'$, we denote it as $k_\varrho(\tau)$.

S2 Appendix: Spherical harmonics and spherical Fourier transform

Spherical harmonics are the generalization of sine and cosine on the surface of a sphere. They are parametrized by the integers l and m, of which l is a positive integer and $m \in \{-l,\ldots,l\}$. These two parameters determine, respectively, the angular frequency and the spatial orientation. Spherical harmonics are defined by the following formula:

$$\mathcal{H}_l^m(\boldsymbol{x}) = \mathcal{H}_l^m(\alpha,\theta) = \sqrt{\frac{(2l+1)(l-|m|)!}{4\pi(l+|m|)!}} P_l^{|m|} \cos\alpha \begin{cases} 1, & \text{for } m=0 \\ \sqrt{2}\cos m\theta & \text{for } m>0 \\ \sqrt{2}\sin|m|\theta & \text{for } m<0 \end{cases}, \tag{9}$$

where $P_l^{|m|}$ is a Legendre polynomial [1].

Spherical harmonics form a set of orthonormal basis functions and, consequently, we can use them to define a spherical Fourier analysis [2]. Specifically, the spatiotemporal process $\alpha(x,t)$ can be expressed as a linear combination of spherical harmonics

$$\alpha(x,t) = \sum_{l,m} \tilde{\alpha}(l,m;t)\mathcal{H}_l^m(\boldsymbol{x}), \tag{10}$$

where $\tilde{\alpha}(l,m;t)$ is the l,m-th spherical Fourier coefficient as a function of time, defined as

$$\tilde{\alpha}(l,m;t) = \int_C \alpha(l,m;t)\mathcal{H}_l^m(\boldsymbol{x})d\boldsymbol{x}. \tag{11}$$

Eqs. (10) and (11) are the equivalent of respectively inverse and direct Fourier transform for functions defined on the surface of a sphere.

S3 Appendix: Properties of the Kronecker product and GP regression with separable covariance matrices

In order to derive the posterior expectations of the spatiotemporal GP regression, it is useful to introduce some of the properties of the Kronecker product between matrices. The Kronecker product between two $N \times N$ matrices is defined by the block form:

$$A \otimes B = \begin{bmatrix} a_{11}B & \cdots & a_{1N}B \\ \vdots & \ddots & \vdots \\ a_{N1}B & \cdots & a_{NN}B \end{bmatrix}. \tag{12}$$

The following formula relates the regular matrix product with the Kronecker product:

$$(A \otimes B)(C \otimes D) = (AC) \otimes (BD) \tag{13}$$

The inverse and transpose of a Kronecker product are respectively

$$(A \otimes B)^{-1} = A^{-1} \otimes B^{-1} \,. \tag{14}$$

and

$$(A \otimes B)^{T} = A^{T} \otimes B^{T} \,. \tag{15}$$

The following formula relates the Kronecker product to the vectorization of a matrix:

$$(A \otimes B)\text{vec}(C) = \text{vec}(B^T C A). \tag{16}$$

Using these formulas, we can now derive the posterior expectation of the spatiotemporal GP regression. Combining the spatiotemporal prior and the observation model using Bayes' theorem, we obtain the posterior

$$p(\text{vec}(\tilde{R})|\text{vec}(Y)) \propto N(\text{vec}(Y)|(\Lambda \otimes I)\text{vec}(\tilde{R}), \Sigma \otimes K_\xi) N(\text{vec}(\tilde{R})|0, D \otimes K_\rho) \tag{17}$$

This is the product of two multivariate Gaussian densities and it is therefore a multivariate Gaussian itself. Its expectation is given by

$$\text{vec}(M_{\tilde{\rho}|y}) = (K_\rho \otimes D)(I \otimes \Lambda)^T \left((I \otimes \Lambda)(K_\rho \otimes D)(I \otimes \Lambda)^T + (K_\xi \otimes \Sigma) \right)^{-1} \text{vec}(Y). \tag{18}$$

Using (13) and (15), the expression simplifies to:

$$\text{vec}(M_{\tilde{\rho}|y}) = \big(K_\rho \otimes (D\Lambda^T)\big) \left(K_\rho \otimes (\Lambda D \Lambda^T) + (K_\xi \otimes \Sigma) \right)^{-1} \text{vec}(Y). \tag{19}$$

This formula involves the inversion of a matrix that is the sum of two Kronecker product components. Inverting this matrix would be computationally impractical. We simplify the problem by imposing $\Sigma = \Lambda D \Lambda^T$. In this case, Eq. (14) allows to invert the spatial and temporal covariance matrices separately:

$$\text{vec}(M_{\tilde{\rho}|y}) = \big(K_\rho \otimes (D\Lambda^T)\big) \left(\big(K_\rho + K_\xi\big)^{-1} \otimes \big(\Lambda D \Lambda^T\big)^{-1} \right) \text{vec}(Y). \tag{20}$$

In most realistic cases, the MEG observation model Λ will not be full rank, therefore we introduced a Tikhonov regularization parameter λ.

$$\big(\Lambda D \Lambda^T\big)^{-1} \;\to\; \big(\Lambda D \Lambda^T + \lambda I\big)^{-1} \tag{21}$$

Using Eq. (16), we finally arrive at the posterior expectation:

$$M_{\tilde{\rho}|Y} = D\Lambda^T (\Lambda D \Lambda^T + \lambda I)^{-1} Y (K_\rho + K_\xi)^{-1} K_\rho \,.$$

S4 Appendix: Modeling vector-valued sources using block matrices

In the main text, the source reconstruction formulae are expressed for fixed dipole directions $\vec{v}(\boldsymbol{x})$. The solution for the general case, in which the dipole direction is estimated from the data, is obtained by introducing an independent set of spherical

harmonics for each of the orthogonal spatial directions $\vec{v}_1$, $\vec{v}_2$, and $\vec{v}_3$. In this Appendix, we refer to the (spherical harmonics domain) forward model matrix relative to the k-th direction as Λ_k. Using this notation, we can define the total forward model matrix with the following block form:

$$\Lambda_{tot} = \begin{bmatrix} \Lambda_1 \\ \Lambda_2 \\ \Lambda_3 \end{bmatrix}. \tag{22}$$

Using an analogous notation, the total spherical harmonics covariance matrix can be written in the following block diagonal form:

$$D_{tot} = \begin{bmatrix} D_1 & 0 & 0 \\ 0 & D_2 & 0 \\ 0 & 0 & D_3 \end{bmatrix}. \tag{23}$$

Hence, the general source reconstruction formula in the spherical harmonics domain is obtained by replacing D and Λ with D_{tot} and Λ_{tot} respectively. This solution can be mapped back to the spatial domain using the total spherical harmonics matrix

$$H_{tot} = \begin{bmatrix} H & 0 & 0 \\ 0 & H & 0 \\ 0 & 0 & H \end{bmatrix}. \tag{24}$$

References

1. Groemer H. Geometric applications of Fourier series and spherical harmonics. vol. 61. Cambridge University Press; 1996.

2. Mohlenkamp MJ. A fast transform for spherical harmonics. J Fourier Anal Appl. 1999;5(2-3):159–184.
